# Supplementary material for: Spatial control of the APC/C ensures the rapid degradation of cyclin B1
Source: EMBO J. 2024 Aug 14;43(19):4324–55. doi: 10.1038/s44318-024-00194-2 (PMC11445581; doi:10.1038/s44318-024-00194-2)
Supplement: Supplementary file 1 — Appendix [file 44318_2024_194_MOESM1_ESM.pdf]

Spatial control of the APC/C ensures the rapid degradation of Cyclin B1

Luca Cirillo*, Rose Young*, Sapthaswaran Veerapathiran, Annalisa Roberti, Molly Martin, Azzah Abubacar, Camilla Perosa, Catherine Coates, Reyhan Muhammad, Theodoros I. Roumeliotis, Jyoti S. Choudhary, Claudio Alfieri+, and Jonathon Pines+

APPENDIX

TABLE OF CONTENT

Appendix Figure S1……………………………………………………………………………pg2
Appendix Figure S2……………………………………………………………………………pg3
Appendix Figure S3……………………………………………………………………………pg4
Appendix Figure S4……………………………………………………………………………pg5
Appendix Figure S5……………………………………………… ………………………..pg6
Appendix Figure S6…………………………………………… ……………………………..pg8

Appendix Table S1……………………………………………………………………………pg10
Appendix Table S2……………………………………………………………………………pg11
Appendix Table S3……………………………………………………………………………pg12

Appendix Figure S1

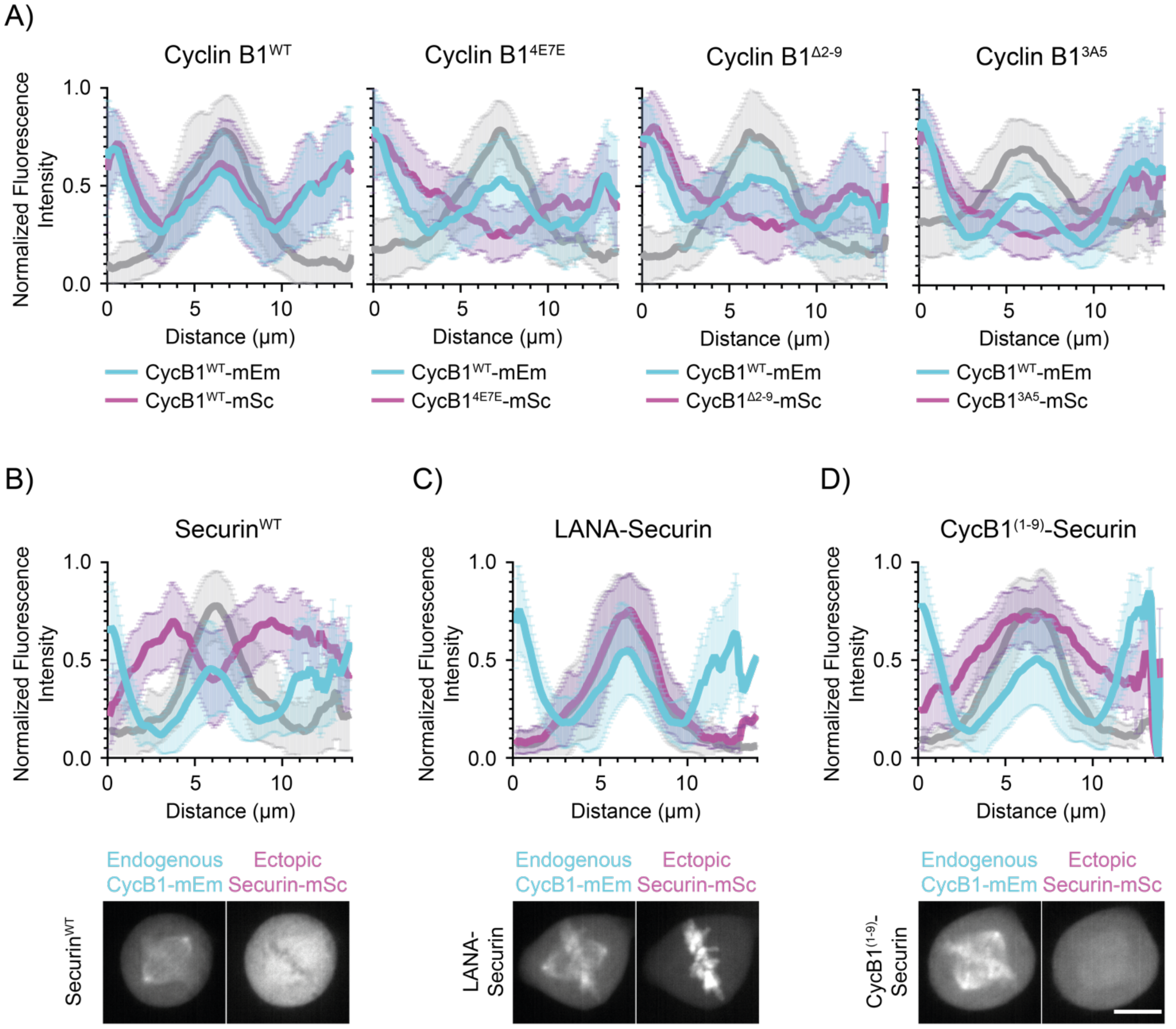

Appendix Figure S1. Subcellular localization of Cyclin B1$^{\Delta 2-9}$ and Cyclin B1^{3A5}

A) Graphs representing the pixel-by-pixel fluorescence intensity along a line going from centrosome to centrosome of RPE-1 Cyclin B1-mEmerald$^{+/+}$ cells ectopically expressing the indicated variant of Cyclin B1-mScarlet. Cyan: endogenous Cyclin B1-mEmerald, Magenta: ectopically expressed Cyclin B1-mScarlet, Grey: siR-DNA: n ≥ 21 cells per condition, N = 3 independent experiments. Mean ± Standard Deviation .

B, C, D) Top: Graphs representing the pixel-by-pixel fluorescence intensity along a line going from centrosome to centrosome of RPE-1 Cyclin B1-mEmerald$^{+/+}$ cells ectopically expressing the indicated variant of Securin-mScarlet. Cyan: endogenous Cyclin B1-mEmerald, Magenta: ectopically expressed Securin-mScarlet, Grey: siR-DNA. Bottom: Maximum projections of representative confocal images used for line profile quantification. Scale bar represents 10 µm. N ≥ 16 cells per condition, N = 3 independent experiments. Mean ± Standard Deviation are plotted.

Appendix Figure S2

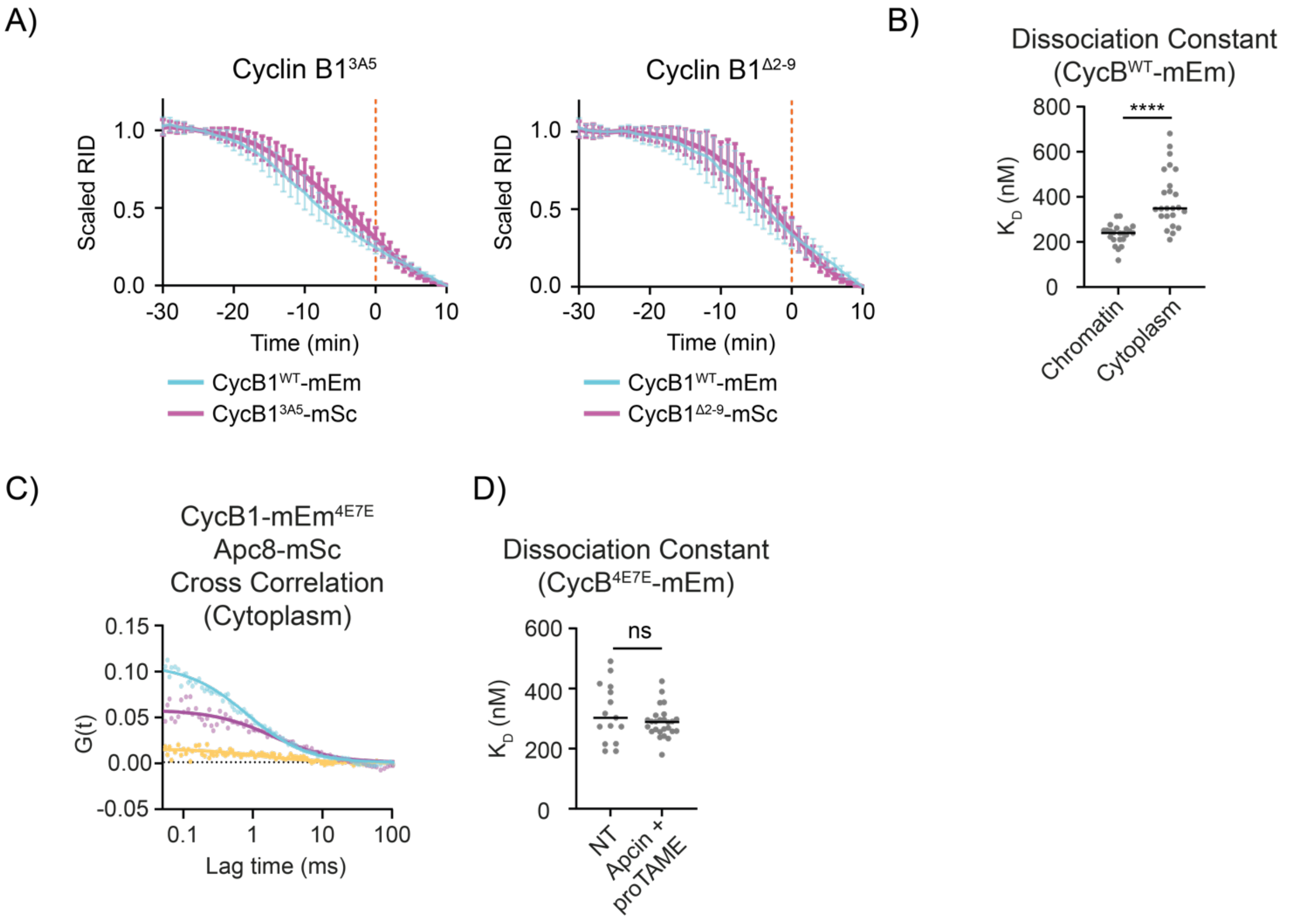

Appendix Figure S2. Degradation of Cyclin B1 mutants and APC8 interaction

A) Cyclin B1 degradation graphs representing the fluorescence intensity of Cyclin B1 over time for cells ectopically expressing the indicated Cyclin B1 variants: n ≥ 16 cells per condition, N ≥ 3 independent experiments. Mean ± Standard Deviation are plotted.

B) Dot plot representing the KD values measured for Cyclin B1 ^{WT}-mEmerald and APC8-mScarlet by FCCS at different subcellular locations: n = 23 cells, N = 3 independent experiments.

C) Representative graph of the autocorrelation function of mEmerald and mScarlet and the cross-correlation function between the two in RPE-1 APC8-mScarlet$^{+/+}$ ectopically expressing Cyclin B1^{4E7E}-mEmerald.

D) Dot plot representing the KD values measured for Cyclin B1 ^{4E7E}-mEmerald and APC8-mScarlet by FCCS before and after a treatment with APCin and proTAME: n ≥ 15 cells, N = 3 independent experiments.

Appendix Figure S3

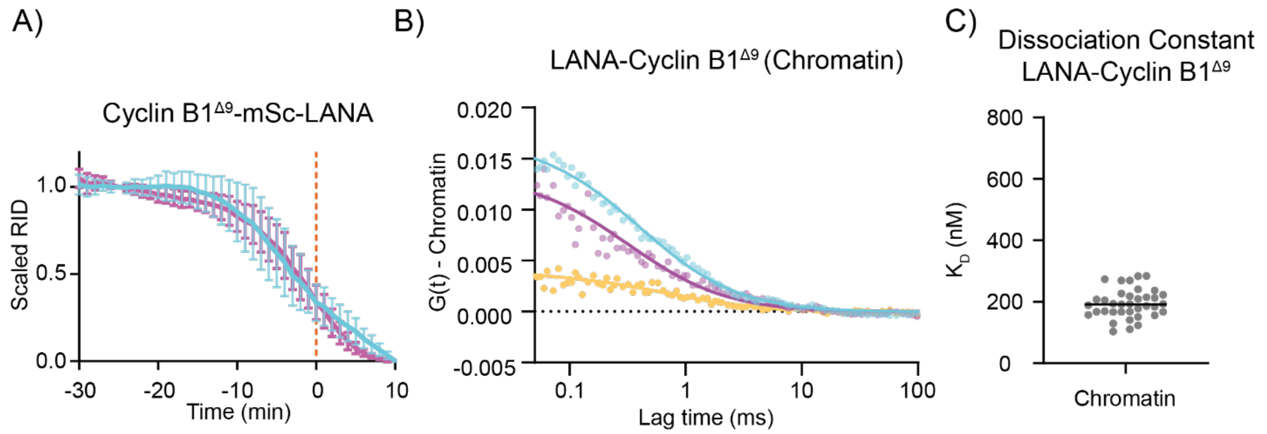

Appendix Figure S3. Degradation of Cyclin B1$^{\Delta9}$-LANA and FCCS of LANA-Cyclin B1$^{\Delta9}$ -mEmerald and APC8-mScarlet interaction.

A) Cyclin B1 degradation graphs representing the fluorescence intensity of Cyclin B1 over time for cells ectopically expressing the indicated Cyclin B1 variant: n = 19 cells, N = 2 independent experiments. Mean ± Standard Deviation are plotted.

B) Representative graph of the autocorrelation function of mEmerald and mScarlet and the cross-correlation function between the two in RPE-1 APC8-mScarlet$^{+/+}$ ectopically expressing LANA-Cyclin B1$^{\Delta9}$-mEmerald.

C) Dot plot representing the KD values measured for LANA-Cyclin B1$^{\Delta9}$-mEmerald and APC8-mScarlet by FCCS in metaphase cells. N = 3 independent experiments.

Appendix Figure S4

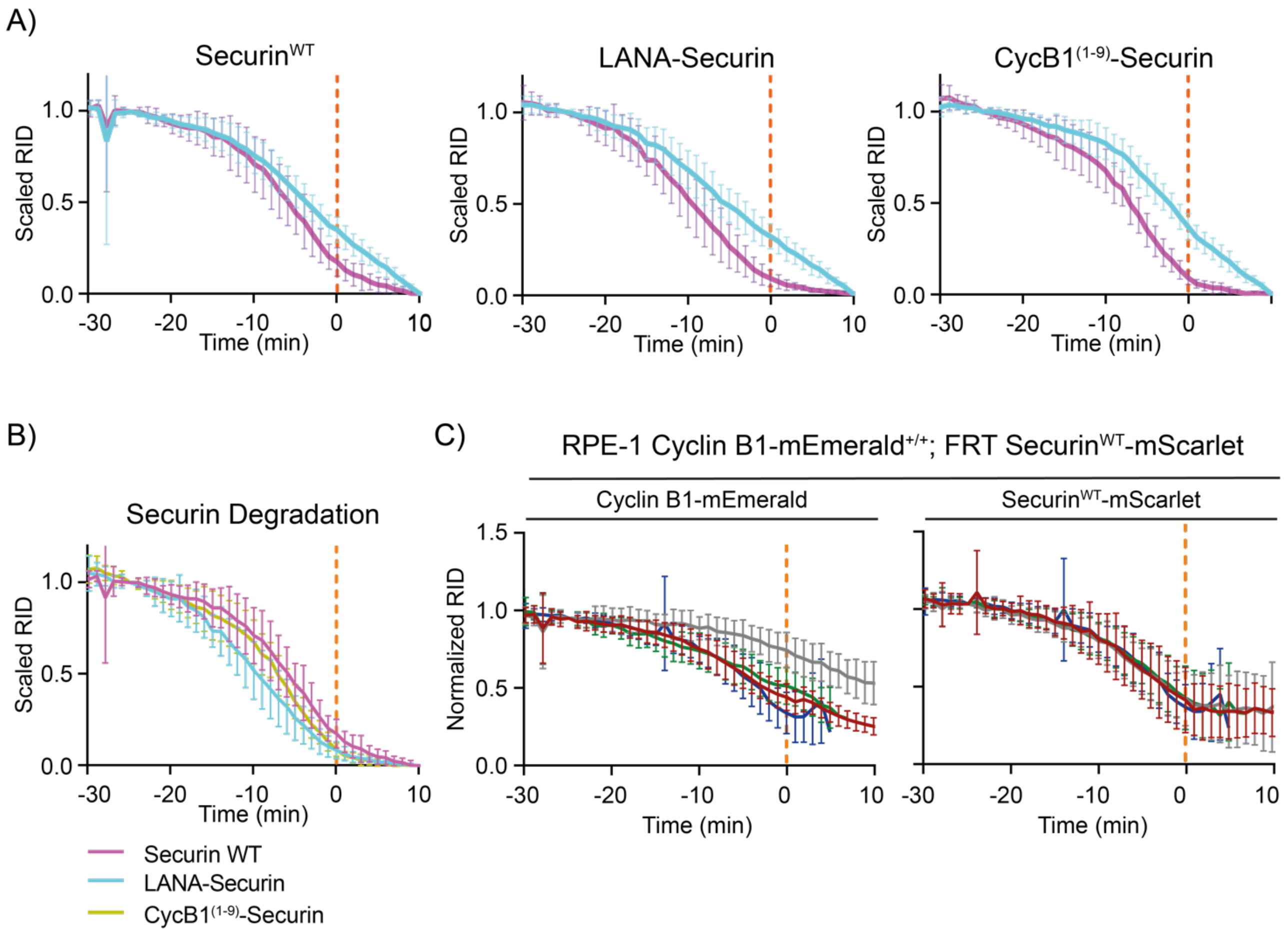

Appendix Figure S4. Localising Securin at the chromatin enhances its degradation

A) Securin degradation graphs representing the fluorescence intensity of Cyclin B1 over time for cells ectopically expressing the indicated Securin variants. Cyan: endogenous Cyclin B1-mEmerald, magenta: ectopic Securin: n ≥ 8 cells per condition, N = 3 independent experiments. Mean ± Standard Deviation are plotted.

B) Securin degradation graph directly comparing the data for Securin degradation shown in A. Mean ± Standard Deviation are plotted.

C) Quantification of normalised Cyclin B1 and Securin fluorescence levels over time measured in RPE-1 CCNB1-mEmerald$^{+/+}$ cells ectopically expressing Securin-mScarlet: n = 12 cells, N = 3 independent experiments. Mean ± Standard Deviation are plotted.

Appendix Figure S5

A)

Clone		TP53 gene	p53 protein
**7A11**	1st allele	G613_614insTGGAGT	E204_Y205insLE
	2nd allele	A578G; G609_610insAAGTTG	H193R; V203_E204insKL
**7H10**	1st allele	G605_606insATTTGCG	V203FfsX8
	2nd allele	T613_614insGGAAATTTGCGTGTGGAGT	Y205WfsX10
**B1H3**	1st allele	G596_597insTGGAAGG	N200GfsX11
	2nd allele	G587_593del; G587_588insGAAATTTGCGTGTGGAGT	V197KfsX17

B)

Clone		TP53 gene	p53 protein
**9B2**	1st allele	G595_598del	G199IfsX47
	2nd allele	A611_612insAATTTGCGTGTGGA	Y205IfsX46
**9B3**	1st allele	T608_609insAAGGAAATTTGCGTGT	E204RfsX9
	2nd allele	G589_595del	R196_E198del

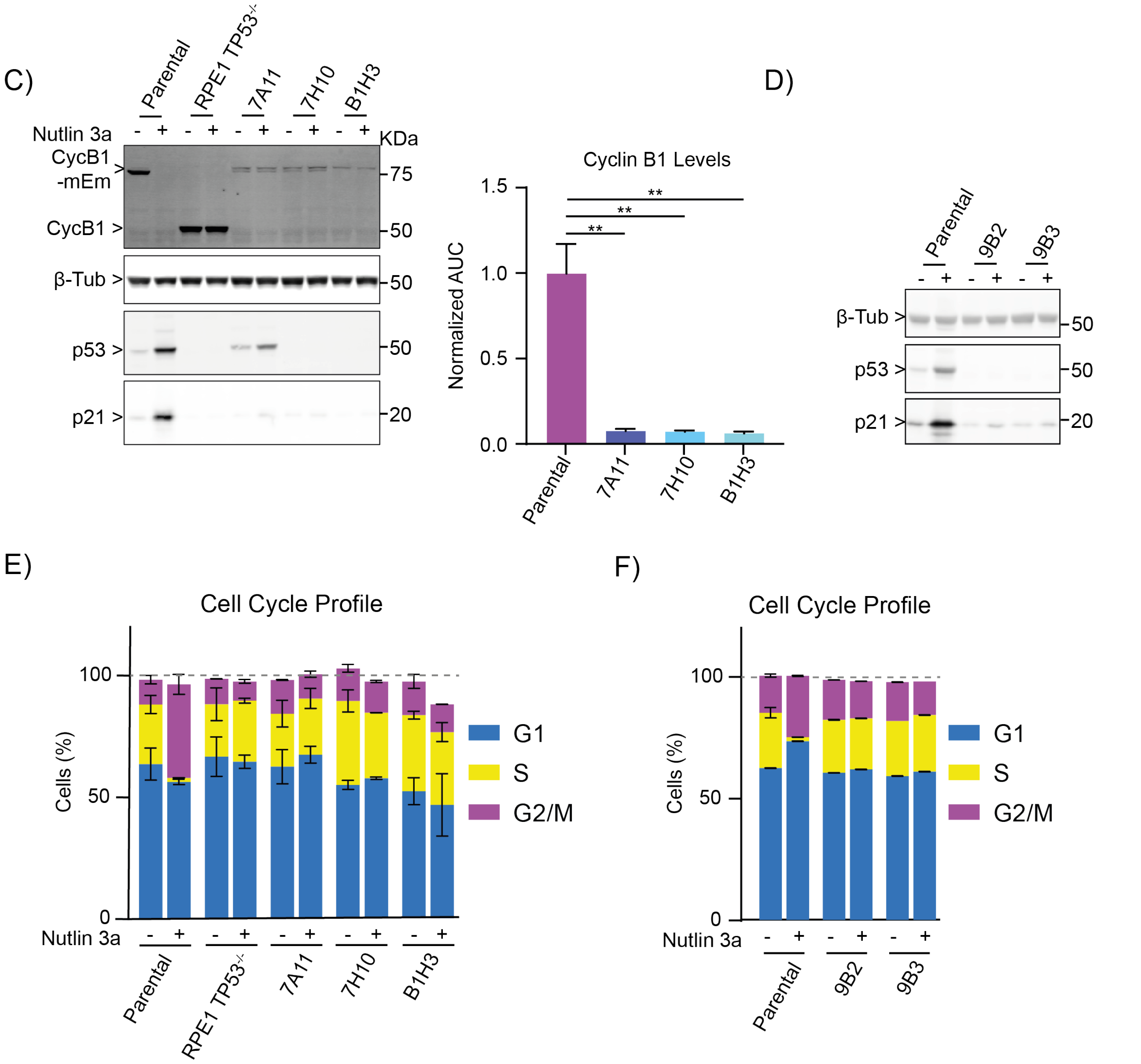

Appendix Figure S5. Characterization of RPE-1 CCNB1^{4E7E}-mEmerald$^{+/+}$; TP53$^{-/-}$ cells

A) Table recapitulating the sequencing of TP53 gene in RPE-1 CCNB1^{4E7E}-mEmerald$^{+/+}$ clones.

B) Table recapitulating the sequencing of TP53 gene in RPE-1 CCNB1-mEmerald$^{+/+}$ clones.

C) Right: representative immunoblot of cell lysates from parental RPE-1 CCNB1-mEmerald$^{+/+}$, RPE-1 TP53$^{-/-}$ or RPE-1 CCNB1^{4E7E}-mEmerald$^{+/+}$ clones, either treated for 24 hr with Nutlin3a, or left untreated. Left: Bar graph representing the quantification of the immunoblot of Cyclin B1. N = 2 independent experiments. Mean ± Standard Deviation.

D) Representative immunoblot of cell lysates from parental RPE-1 CCNB1-mEmerald$^{+/+}$ or RPE-1 CCNB1-mEmerald$^{+/+}$; TP53$^{-/-}$ clones, either treated for 24h with Nutlin3a, or left untreated.

E) Bar graph representing the quantification of the cell cycle profiles of parental RPE-1 CCNB1-mEmerald$^{+/+}$, RPE-1 TP53$^{-/-}$ or RPE-1 CCNB1^{4E7E}-mEmerald$^{+/+}$ clones, either treated for 24h with Nutlin3a, or untreated. N = 2 independent experiments. Mean ± Range.

F) Bar graph representing the quantification of the cell cycle profiles of parental RPE-1 CCNB1-mEmerald$^{+/+}$ or RPE-1 CCNB1-mEmerald$^{+/+}$; TP53$^{-/-}$ clones, either treated for 24h with Nutlin3a, or left untreated. N = 2 independent experiments. Mean ± Range.

Appendix Figure S5 – Supplementary text

Nutlin-3A increased p21 levels in the parental cells, and Cyclin B1 levels decreased as a consequence of cell cycle arrest. We did not detect significant changes in p21 or Cyclin B1 levels in any of the 4E7E p53$^{-/-}$ mutant clones, nor in control RPE-1 TP53$^{-/-}$ cells (Chiang et al., 2019) (Appendix Fig. S5C, D). Flow cytometry analysis of PI-stained cells indicated a robust accumulation of 2n and 4n parental cells following Nutlin-3A treatment, indicative of a p53-dependent cell cycle arrest, whereas RPE-1 TP53$^{-/-}$, Cyclin B1^{4E7E} clones and TP53$^{-/-}$ clones displayed no signs of cell cycle arrest (Appendix Fig. S5E, F). In unperturbed conditions, Cyclin B1^{4E7E} clones and TP53$^{-/-}$ clones did not show any significant variation in cell cycle progression (Appendix Fig. S5E, F).

Appendix Figure S6

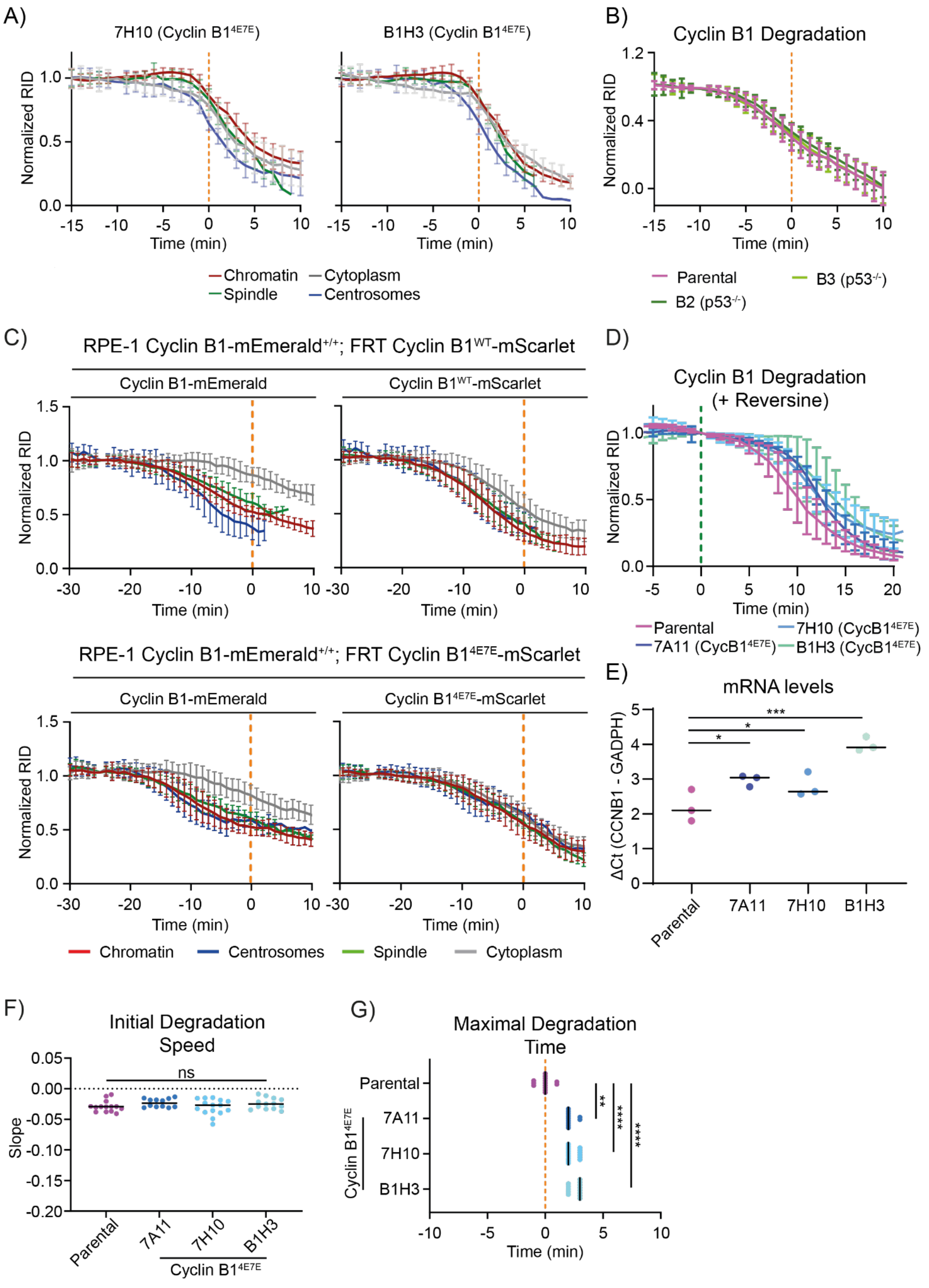

Appendix Figure S6. Degradation analysis of Cyclin B1^{4E7E}

A, B) Cyclin B1 degradation graphs representing the fluorescence intensity of Cyclin B1 over time of RPE-1 Cyclin B1-mEmerald$^{+/+}$ compared to Cyclin B1^{4E7E} clones or p53$^{-/-}$ clones. Mean ± Standard Deviation are plotted.

C) Quantification of normalised Cyclin B1 fluorescence levels over time measured by spinning disk fluorescence microscopy in RPE-1CCNB1-mEmerald$^{+/+}$ cells ectopically expressing the indicated Cyclin-mScarlet B1 variant: n ≥ 18 cells per condition, N = 3 independent experiments. Mean ± Standard Deviation are plotted.

D) Cyclin B1 degradation graph representing the fluorescence intensity of Cyclin B1 over time of RPE-1 Cyclin B1-mEmerald$^{+/+}$ compared to Cyclin B1^{4E7E} clones or p53$^{-/-}$ clones, following exposure to Reversine. Green dotted line indicates nuclear envelope breakdown. Mean ± Standard Deviation are plotted.

E) Dot plots representing the mRNA levels in RPE-1 Cyclin B1-mEmerald$^{+/+}$ compared to Cyclin B1^{4E7E} clones. Ct = Cycle threshold.

F, G) Dot plots representing the initial degradation speed (D) or the maximal degradation time (E) of RPE-1 Cyclin B1-mEmerald$^{+/+}$ compared to Cyclin B1^{4E7E} clones.

Appendix Table S1

**Subcellular Location**	**Concentration (apparent) of CyclinB1-mEmerald**	**Concentration (apparent) of APC8-mScarlet**	**Dissociation Constant (apparent)**
Chromosomes	120 ± 13 nM	103 ± 10 nM	83 ± 40 nM
Cytoplasm	59 ± 8.5 nM	99 ± 12 nM	176 ± 62 nM
Spindle Fibres	92 ± 7.5 nM	103 ± 11 nM	71 ± 31 nM
Spindle Poles	141 ± 17 nM	98 ± 11 nM	81 ± 19 nM

Appendix Table S1. Concentrations of Cyclin B1-mEmerald and APC8-mScarlet at different subcellular locations. Mean ± Standard Deviation.

Appendix Table S2

Figure	Mean difference (p value)		Multiple comparison (p value)		Stastical Test
Fig 1H	<0.0001		Cytoplasm vs. Chromatin	<0.0001	Kruskal-Wallis; Dunn's Multiple Comparison
			Cytoplasm vs. Spindle	<0.0001	
			Cytoplasm vs. Centrosomes	<0.0001	
Fig 1I	0.3435				one-way ANOVA
Fig 1J	0.2348				unpaired t-test
Fig 7E	<0.0001		Parental vs 7A11	<0.0001	Welch's ANOVA; Dunn's Multiple Comparison
			Parental vs 7H10	<0.0001	
			Parental vs B1H3	<0.0001	
Fig 7F	0.075				Kruskal-Wallis
Fig 7G	<0.0001		Parental vs 7A11	0.016	Kruskal-Wallis; Dunn's Multiple Comparison
			Parental vs 7H10	<0.0001	
			Parental vs B1H3	<0.0001	
Fig S1C	top left	0.936			one-way ANOVA
	top right	0.629			one-way ANOVA
	bottom left	0.304			one-way ANOVA
	bottom right	0.464			one-way ANOVA
Fig S1D	top	0.797			two-way ANOVA
	bottom	0.9201			Kruskal-Wallis
Fig S1F	left	0.379			one-way ANOVA
	middle	<0.001			one-way ANOVA
	right	0.2			Kruskal-Wallis
Fig S2D	<0.0001		Cytoplasm vs. Chromatin	<0.0001	Kruskal-Wallis; Dunn's Multiple Comparison
			Cytoplasm vs. Spindle	<0.0001	
			Cytoplasm vs. Centrosomes	<0.0001	
Fig S6B	<0.0001				unpaired t-test
Fig S6D	0.1952				unpaired t-test
Fig S9C	0.001		Parental vs. 7A11	0.0016	one-way ANOVA; Tukey's Multiple Comparison
			Parental vs. 7H10	0.0016	
			Parental vs. B1H3	0.0016	
Fig S10E	0.0009		Parental vs 7A11	0.0356	one-way ANOVA; Tukey's Multiple Comparison
			Parental vs 7H10	0.0469	
			Parental vs B1H3	0.0004	
Fig S10F	0.234				Kruskal-Wallis
Fig S10G	<0.0001		Parental vs 7A11	0.01	Kruskal-Wallis; Dunn's Multiple Comparison
			Parental vs 7H10	<0.0001	
			Parental vs B1H3	<0.0001	

Appendix Table S2. Statistical analysis used through the study.

Appendix Table S3

	NCP-CbNT	NCP-APC3 loop
**Data collection and processing**		
Magnification	150,000	165,000
Voltage (kV)	200	300
Electron exposure (e–/Å2)	60	62
Defocus range (μm)	-0.6 to -1.6	-0.6 to -1.6
Pixel size (Å)	0.94	0.52
Symmetry imposed	C1	C1
Initial particle images (no.)	1,201,152	2,698,450
Final particle images (no.)	38,352	414,277
Map resolution (Å)	2.5	2.5
FSC threshold	0.143	0.143
**Refinement**		
Initial model used (PDB code)	3LZ0	9FH9
Model resolution (Å)	2.5	2.5
Map sharpening B factor (Å2)	-22	-50
Model composition		
Nonhydrogen atoms	11862	11519
Protein residues	747	762
Nucleotides	290	290
B factors (Å2)		
Protein (min/max/mean)	9.44/86.92/36.71	10.06/85.06/34.16
Nucleotide (min/max/mean)	13.40/206.15/84.84	29.26/133.94/69.73
R.m.s. deviations		
Bond lengths (Å)	0.004	0.003
Bond angles (°)	0.627	0.564
**Validation**		
MolProbity score	2	1.19
Clashscore	8.76	3.99
Poor rotamers (%)	3.23	0.94
Ramachandran plot		
Favored (%)	97.25	98.52
Allowed (%)	2.75	1.48
Disallowed (%)	0	0

Appendix Table S3. Cryo-EM data collection, refinement and validation statistics
