# Supplementary material for: Spatial control of the APC/C ensures the rapid degradation of cyclin B1
Source: EMBO J. 2024 Aug 14;43(19):4324–55. doi: 10.1038/s44318-024-00194-2 (PMC11445581; doi:10.1038/s44318-024-00194-2)
Supplement: Supplementary file 3 — Movie EV1 [file 44318_2024_194_MOESM3_ESM.zip › Movie EV1/Movie EV1 Legend.docx]

**Movie EV1. Spatial patterning of Cyclin B1-mEmerald disappearance at mitotic exit.** Maximum projection of spinning disk confocal time series of of RPE-1 CyclinB1-mEmerald^+/+^ cells. Left: Cyclin B1-mEmerald, right: siR-DNA, right: merge. Time is expressed as hh:mm:ss. Scale bar correspond to 10 μm.
