# Supplementary figures and images for: Spatial control of the APC/C ensures the rapid degradation of cyclin B1
Source: EMBO J. 2024 Aug 14;43(19):4324–55. doi: 10.1038/s44318-024-00194-2 (PMC11445581; doi:10.1038/s44318-024-00194-2)

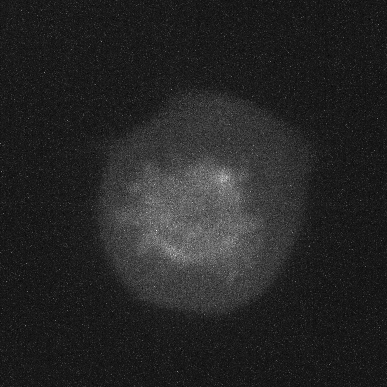

Supplement: Supplementary file 5 — Source data Fig. 1 [file 44318_2024_194_MOESM5_ESM.zip › Figure 1/Fig 1A/200206_Max_NT_pos2.tif]

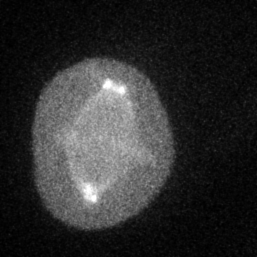

Supplement: Supplementary file 6 — Source data Fig. 2 [file 44318_2024_194_MOESM6_ESM.zip › Figure 2/Fig 2A/MAX_CEi50nM_Rev10uM_B.sld - 221007 - Position 2-1-1.tif]

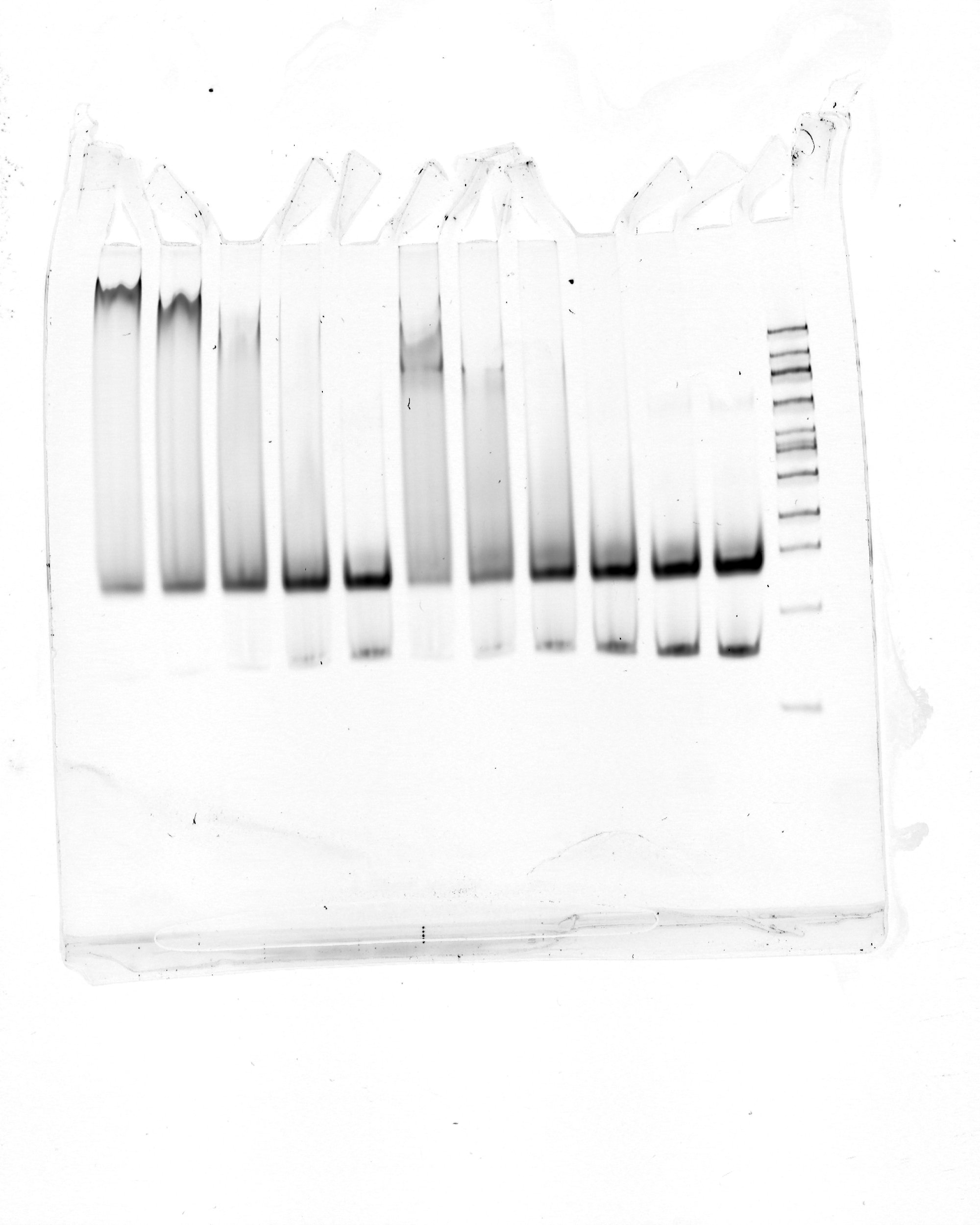

Supplement: Supplementary file 7 — Source data Fig. 3 [file 44318_2024_194_MOESM7_ESM.zip › Figure 3/3E/20240308_APC3loopwtvs3r3e_run2-[SYBR Safe].jpg]

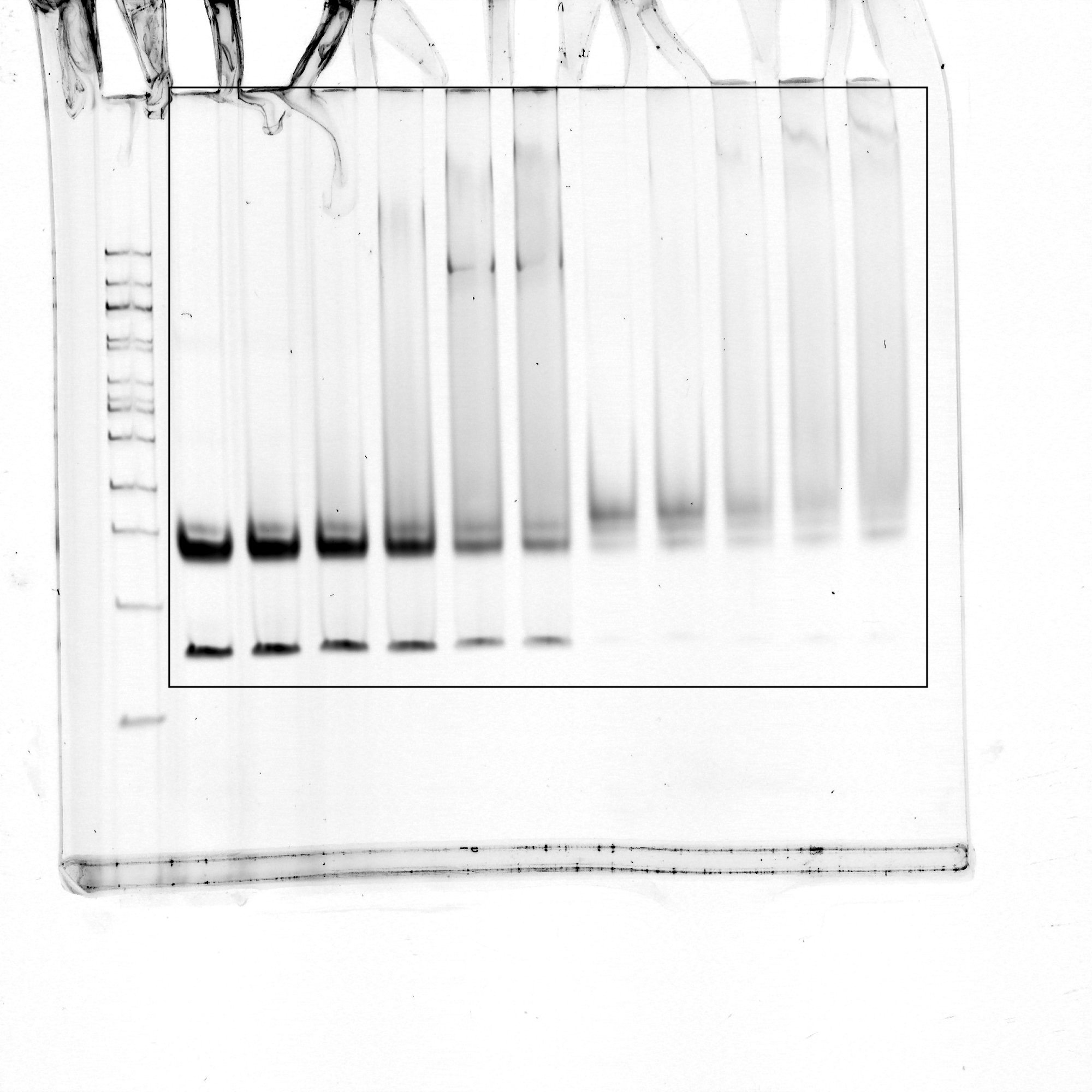

Supplement: Supplementary file 7 — Source data Fig. 3 [file 44318_2024_194_MOESM7_ESM.zip › Figure 3/3E/20240307_APC3loopwtvslana_run2-[SYBR Safe].jpg]

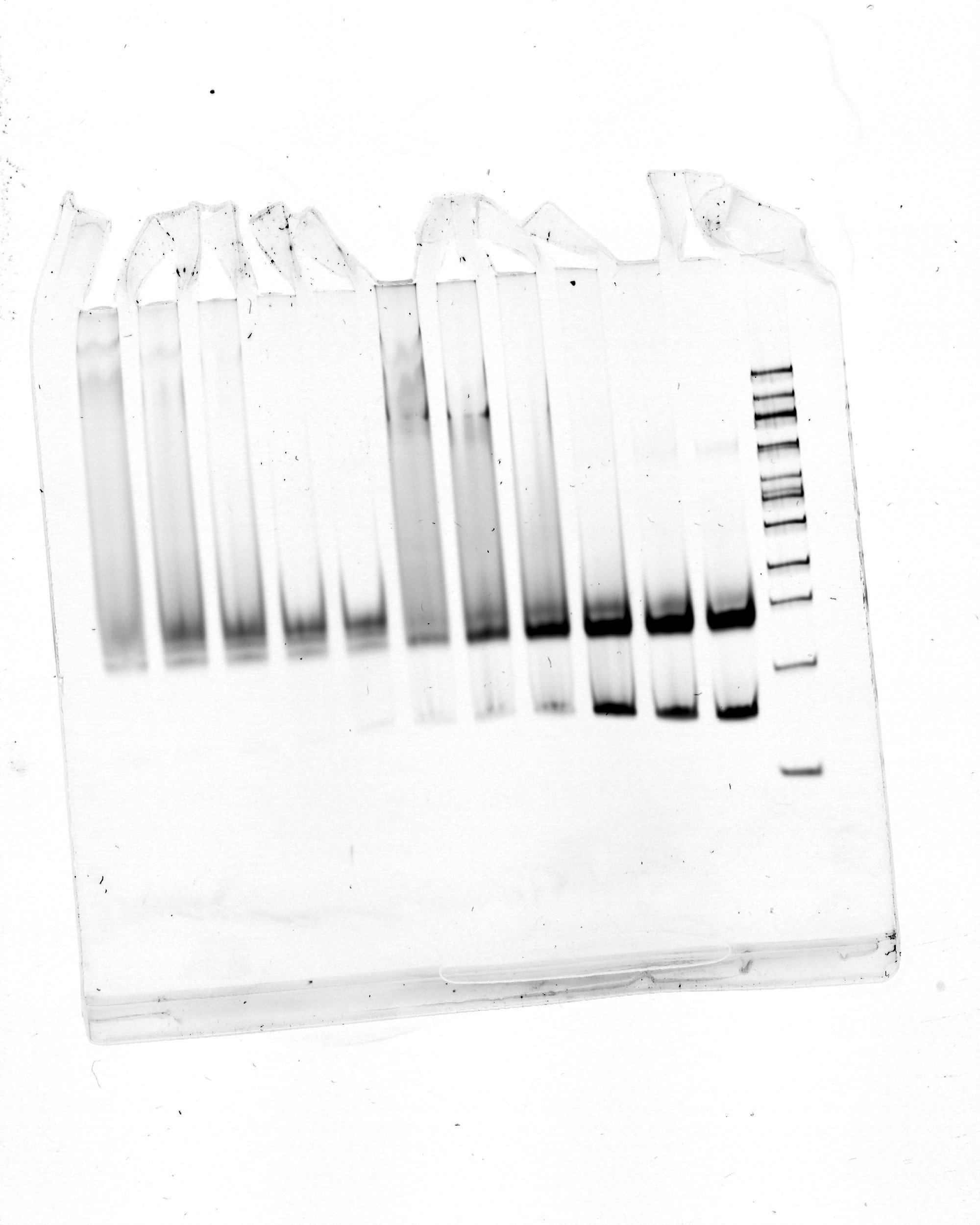

Supplement: Supplementary file 7 — Source data Fig. 3 [file 44318_2024_194_MOESM7_ESM.zip › Figure 3/3E/20240308_wtvslana.jpg]

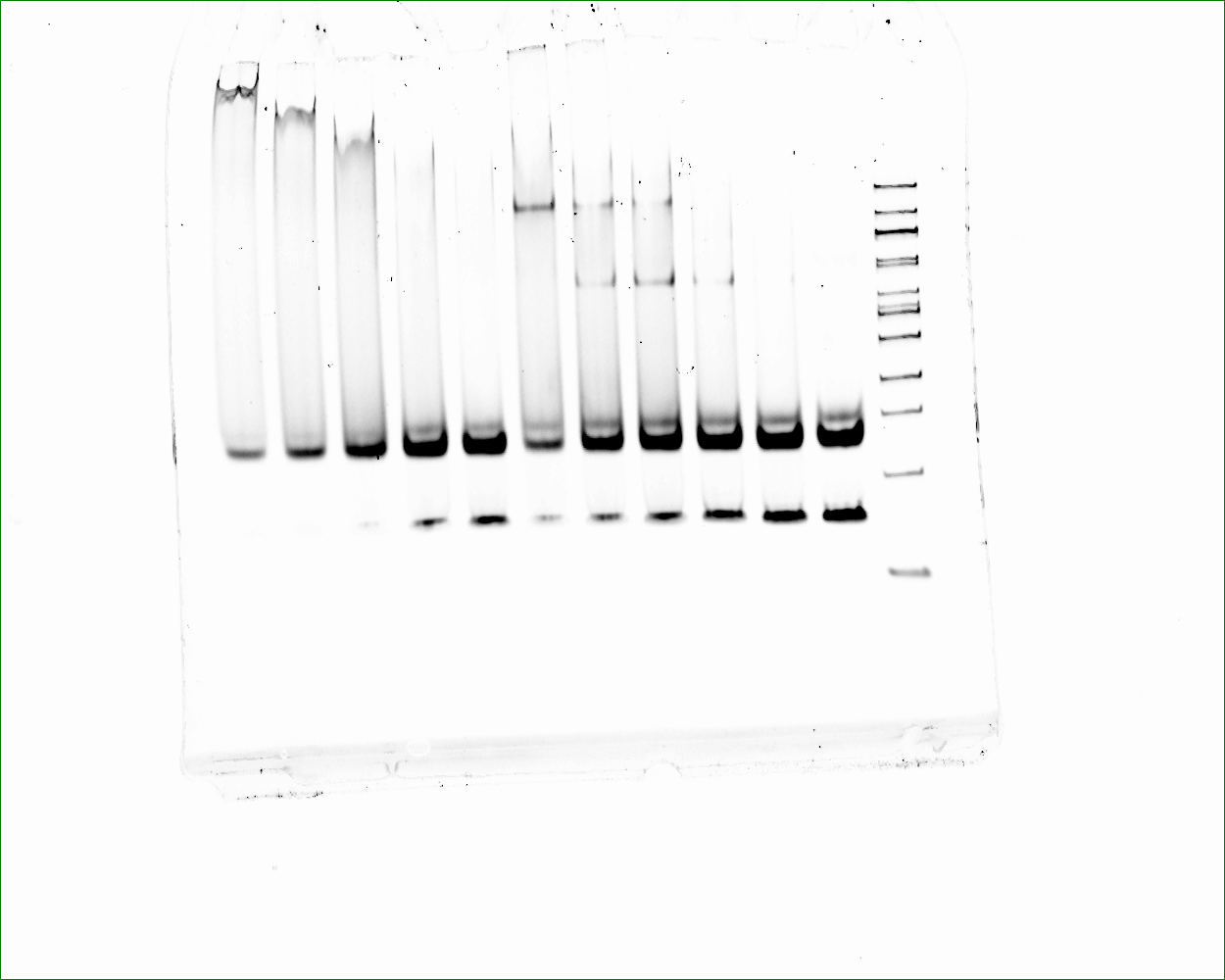

Supplement: Supplementary file 7 — Source data Fig. 3 [file 44318_2024_194_MOESM7_ESM.zip › Figure 3/3D/20240223_wtvs3r3e0.2umNCP.bmp]

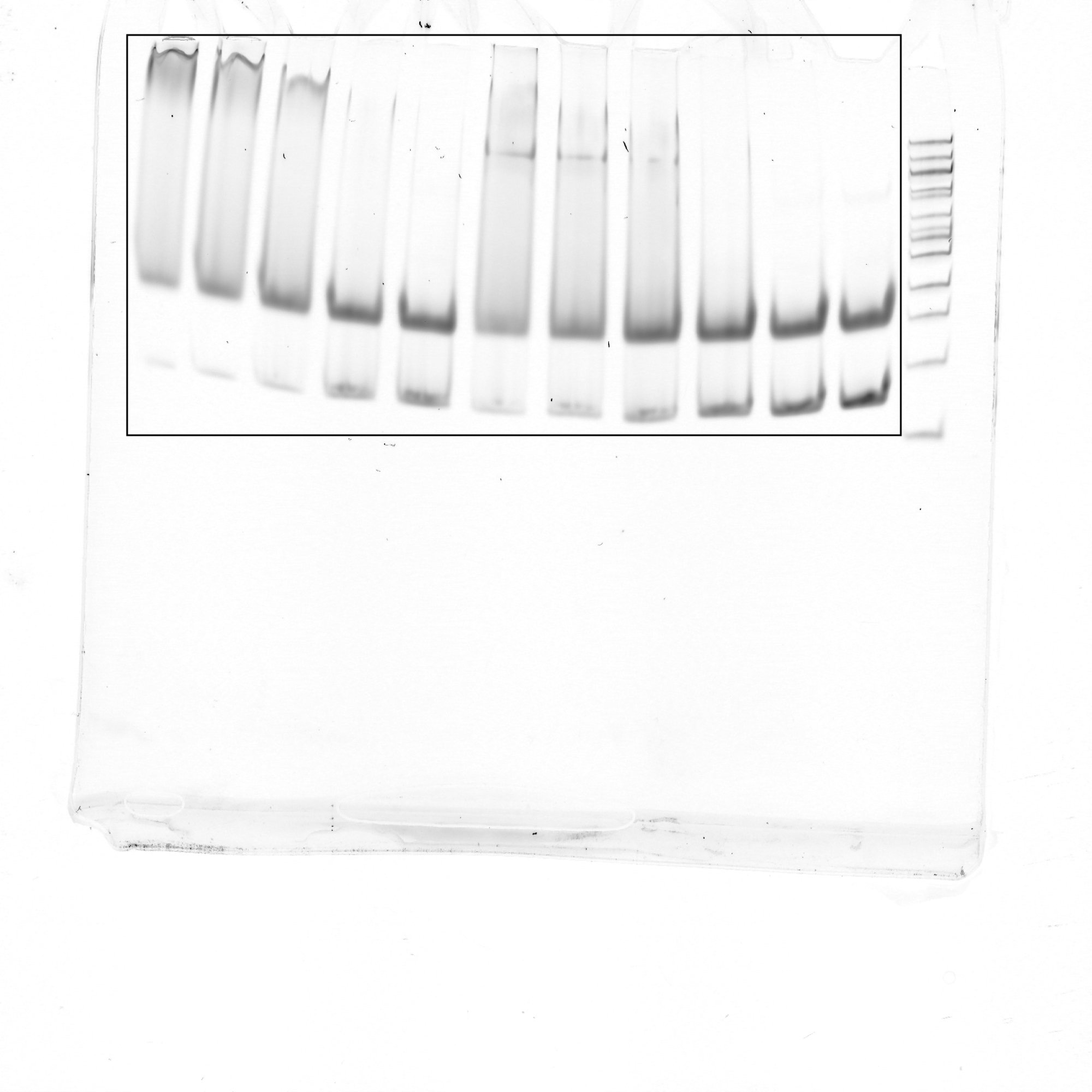

Supplement: Supplementary file 7 — Source data Fig. 3 [file 44318_2024_194_MOESM7_ESM.zip › Figure 3/3D/20240307_APC3loopwtvs3r3e-[SYBR Safe].jpg]

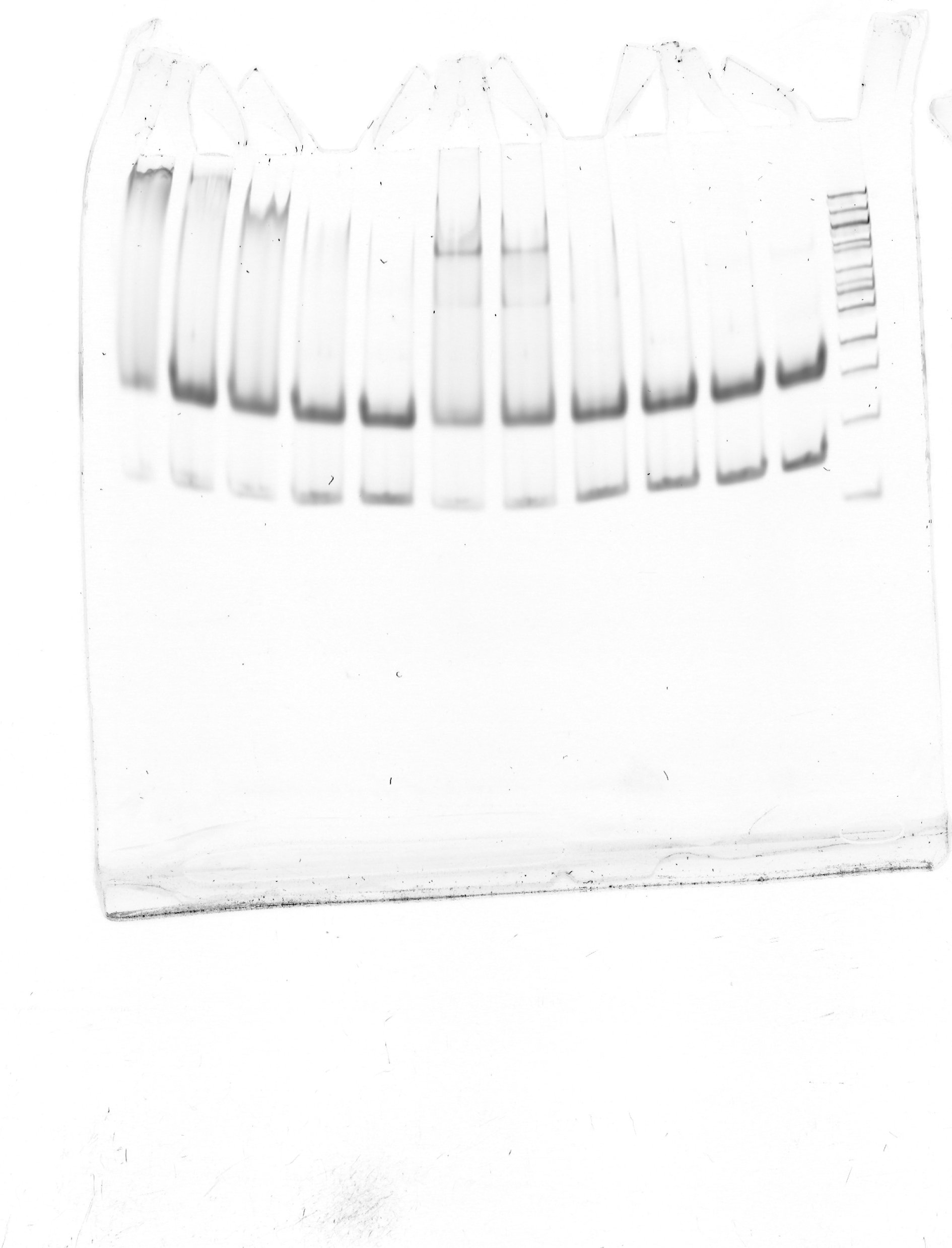

Supplement: Supplementary file 7 — Source data Fig. 3 [file 44318_2024_194_MOESM7_ESM.zip › Figure 3/3D/20240306_APC3loopwtvs3r3e(2repeats)-[SYBR Safe].jpg]

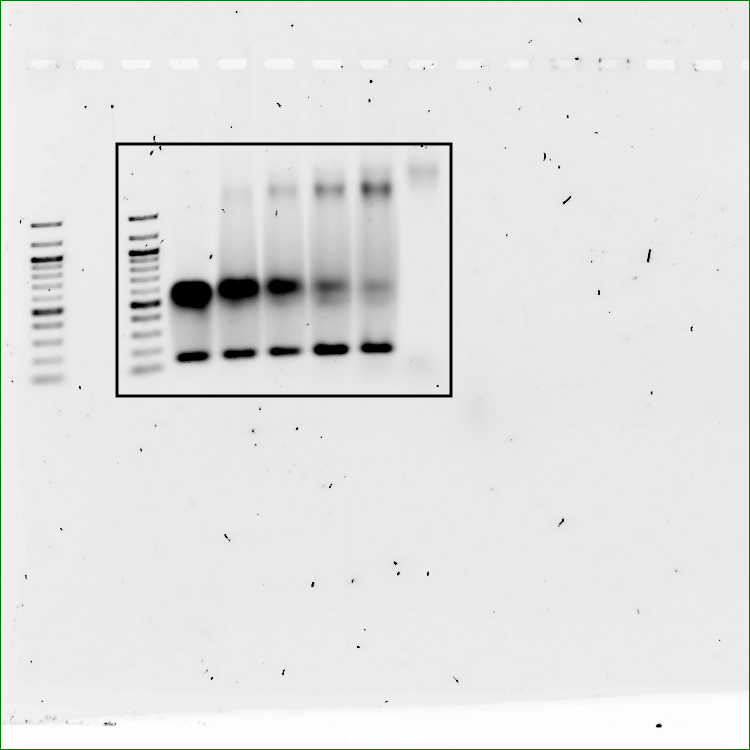

Supplement: Supplementary file 7 — Source data Fig. 3 [file 44318_2024_194_MOESM7_ESM.zip › Figure 3/3A/EMSA NCP with APCCcdc20-cycBNTD.png]

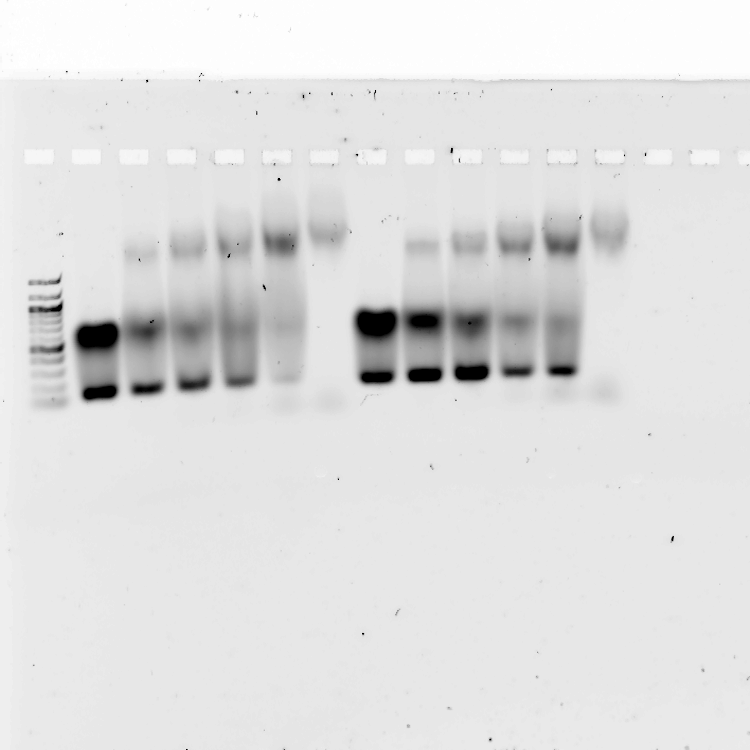

Supplement: Supplementary file 7 — Source data Fig. 3 [file 44318_2024_194_MOESM7_ESM.zip › Figure 3/3A/Replicate EMSA NCP with APCCcdc20-cycBNTD.bmp]

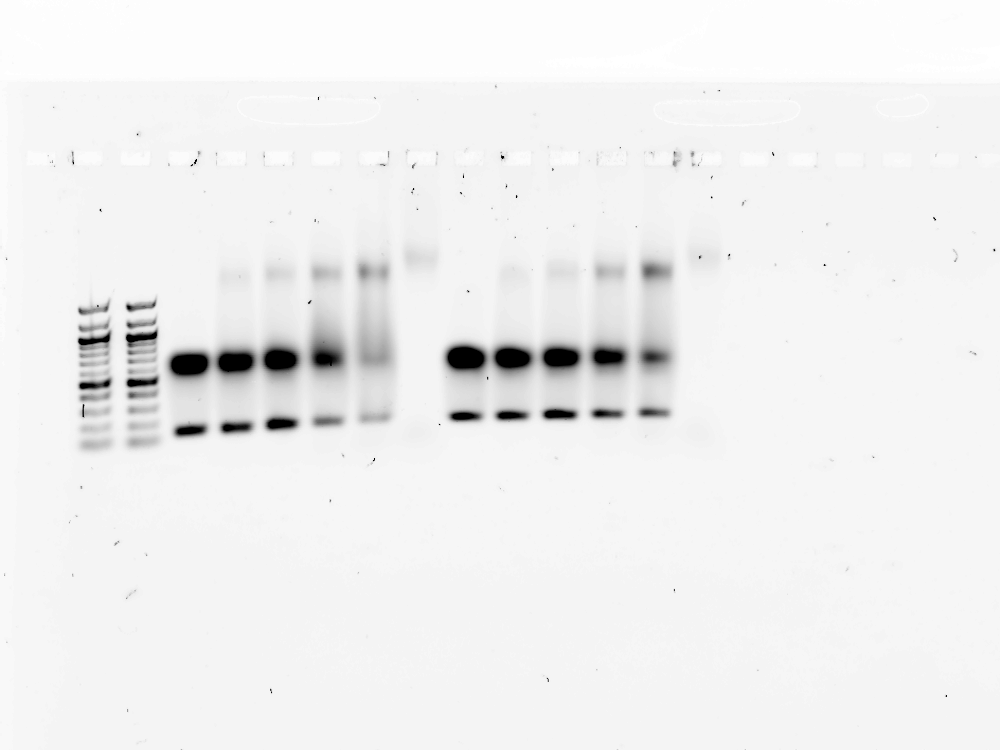

Supplement: Supplementary file 7 — Source data Fig. 3 [file 44318_2024_194_MOESM7_ESM.zip › Figure 3/3A/Replicate2 EMSA NCP with APCCcdc20-cycBNTD.bmp]

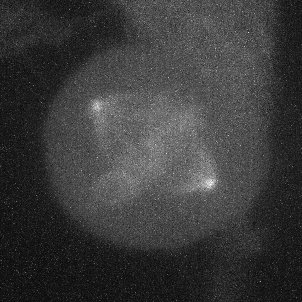

Supplement: Supplementary file 8 — Source data Fig. 4 [file 44318_2024_194_MOESM8_ESM.zip › Figure 4/Fig 4H/MAX_4643.sld - 4643_Mg132 - 6-1.tif]

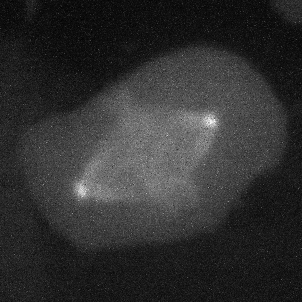

Supplement: Supplementary file 8 — Source data Fig. 4 [file 44318_2024_194_MOESM8_ESM.zip › Figure 4/Fig 4H/MAX_4646.sld - 4646_Mg132 - 1-1.tif]

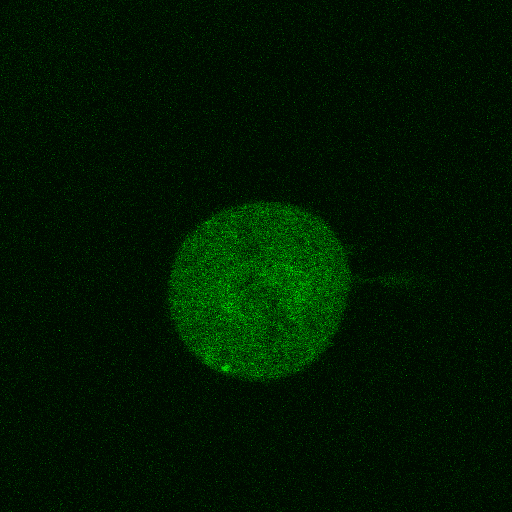

Supplement: Supplementary file 9 — Source data Fig. 5 [file 44318_2024_194_MOESM9_ESM.zip › Figure 5/Fig5 CD/CycB4E7E/4819 Cyclin B1Image006_ch0.png]

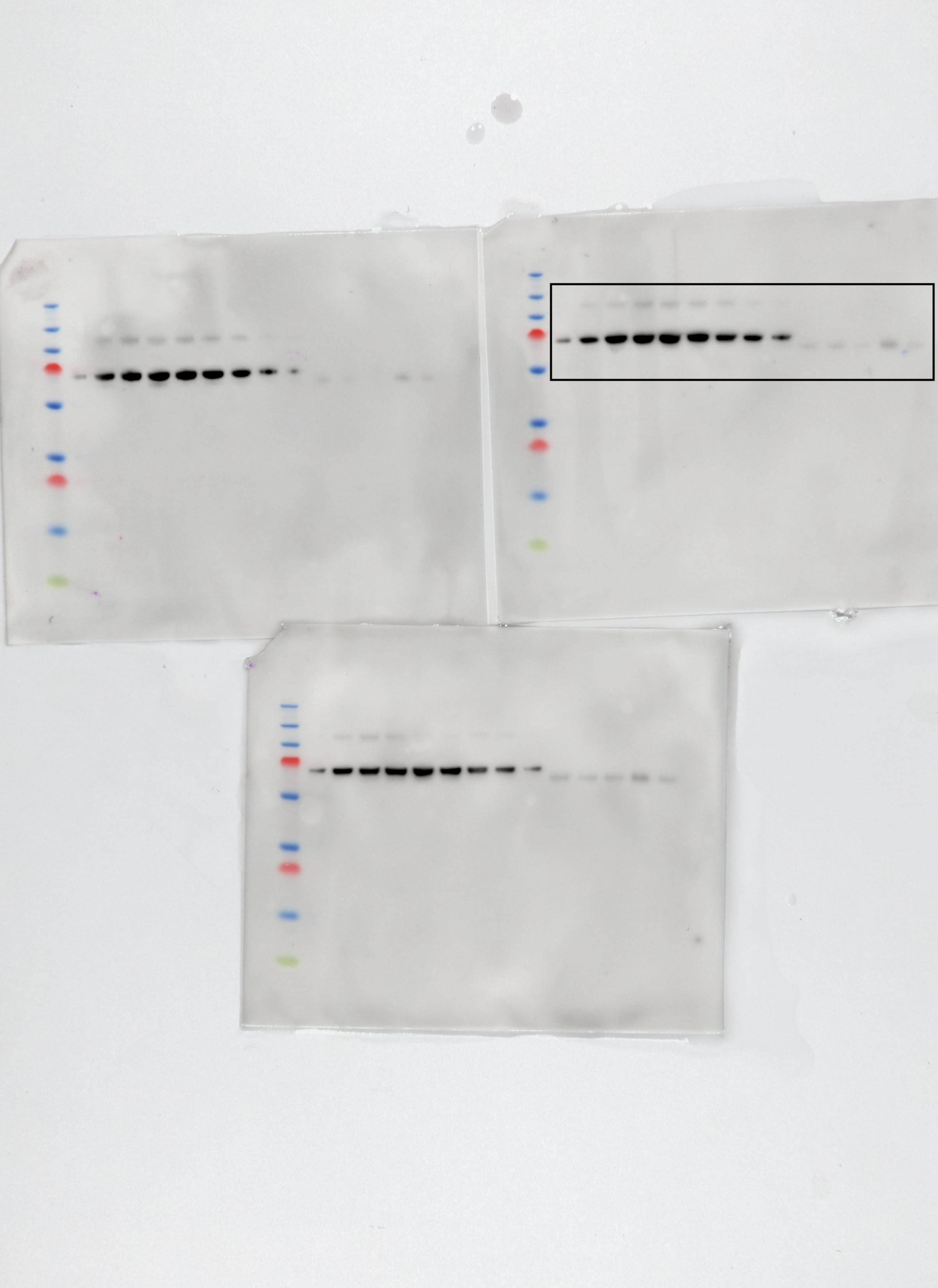

Supplement: Supplementary file 10 — Source data Fig. 6 [file 44318_2024_194_MOESM10_ESM.zip › Figure 6/Fig 6AB/WT cyclin B1 and APC2 western blot.tif]

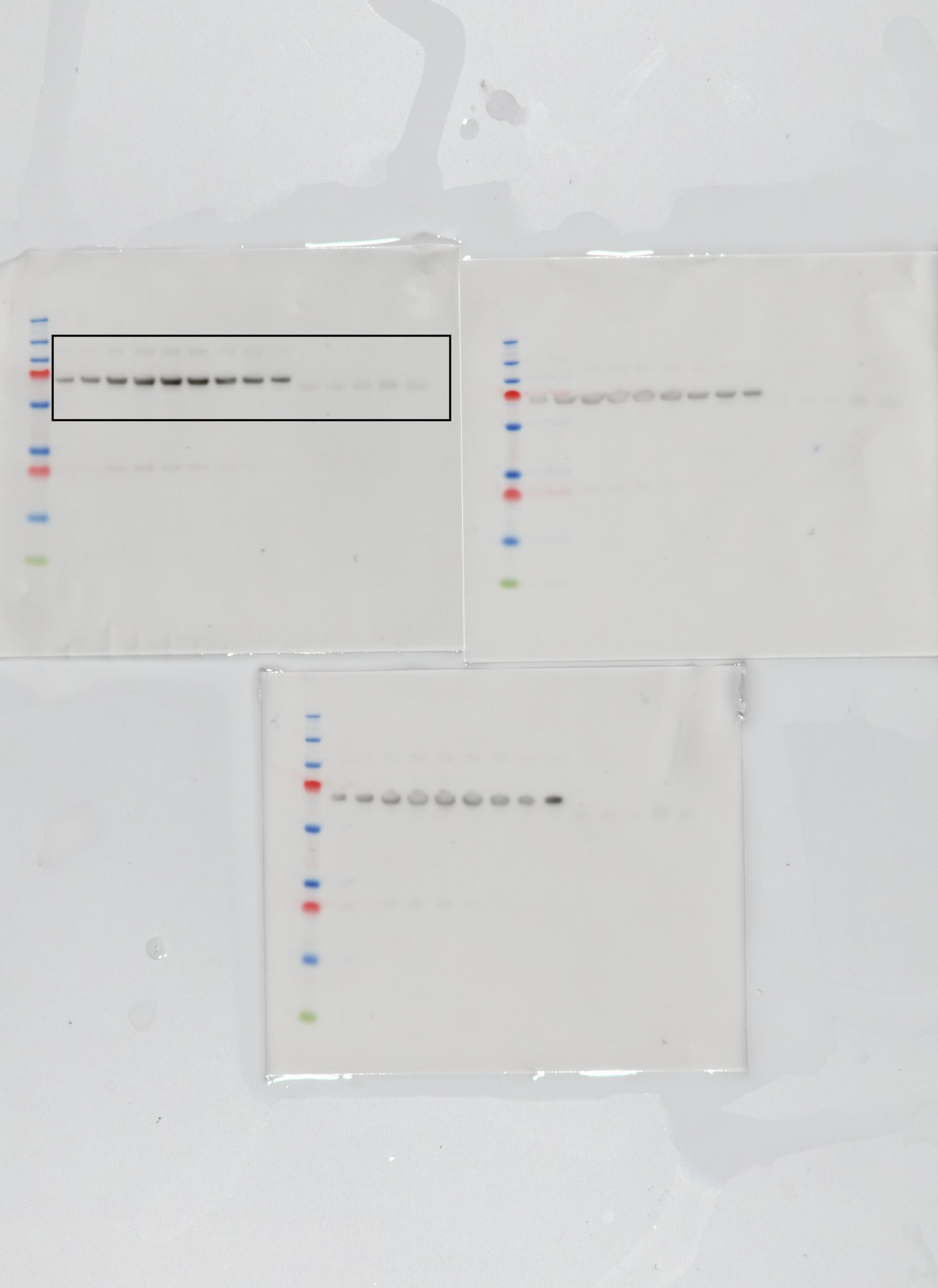

Supplement: Supplementary file 10 — Source data Fig. 6 [file 44318_2024_194_MOESM10_ESM.zip › Figure 6/Fig 6AB/D9 cyclin B1 and APC2 western blot.tif]

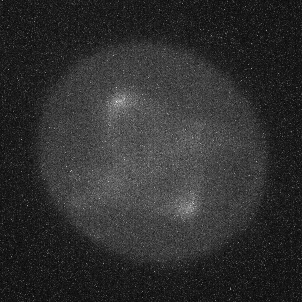

Supplement: Supplementary file 10 — Source data Fig. 6 [file 44318_2024_194_MOESM10_ESM.zip › Figure 6/Fig 6D/MAX_220717.sld - 220617_4816 - 5-1.tif]

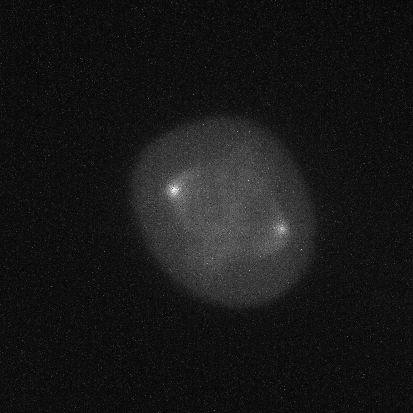

Supplement: Supplementary file 10 — Source data Fig. 6 [file 44318_2024_194_MOESM10_ESM.zip › Figure 6/Fig 6C/MAX_4740.sld - 211014 - Position 1-1.tif]
