# Supplementary material for: Spatial control of the APC/C ensures the rapid degradation of cyclin B1
Source: EMBO J. 2024 Aug 14;43(19):4324–55. doi: 10.1038/s44318-024-00194-2 (PMC11445581; doi:10.1038/s44318-024-00194-2)
Supplement: Supplementary file 8 — Source data Fig. 4 [file 44318_2024_194_MOESM8_ESM.zip › Figure 4/Fig 4I/README.rtf]

4643 = CycB1-mEm+/+ FRT CycB1WT-mSc4646 = CycB1-mEm+/+ FRT CycB14E7E-mScC1 = mEmeraldC2 = mScarletC3 = siR-DNA
