# Supplementary material for: Spatial control of the APC/C ensures the rapid degradation of cyclin B1
Source: EMBO J. 2024 Aug 14;43(19):4324–55. doi: 10.1038/s44318-024-00194-2 (PMC11445581; doi:10.1038/s44318-024-00194-2)
Supplement: Supplementary file 9 — Source data Fig. 5 [file 44318_2024_194_MOESM9_ESM.zip › Figure 5/Fig5 CD/README.rtf]

4817 = APC8-mSc+/+ FRT CycB1WT-mEm4819 = APC8-mSc+/+ FRT CycB14E7E-mEm
