# Supplementary material for: Spatial control of the APC/C ensures the rapid degradation of cyclin B1
Source: EMBO J. 2024 Aug 14;43(19):4324–55. doi: 10.1038/s44318-024-00194-2 (PMC11445581; doi:10.1038/s44318-024-00194-2)
Supplement: Supplementary file 10 — Source data Fig. 6 [file 44318_2024_194_MOESM10_ESM.zip › Figure 6/Fig 6C/README.rtf]

4740 = CycB1-mEm+/+ FRT LANA-d9CycB1-mScC1 = mEmeraldC2 = mScarletC3 = siR-DNANote some samples were wrongly labelled 4640.
