# Supplementary material for: Spatial control of the APC/C ensures the rapid degradation of cyclin B1
Source: EMBO J. 2024 Aug 14;43(19):4324–55. doi: 10.1038/s44318-024-00194-2 (PMC11445581; doi:10.1038/s44318-024-00194-2)
Supplement: Supplementary file 11 — Source data Fig. 7 [file 44318_2024_194_MOESM11_ESM.zip › Figure 7/Sequencing/README.rtf]

CycB16146 (FW) 5’-GCCTTTCATGAACTATATTATTG-3’6173 (FW) 5’-CTGGAAACGCATTCTCTG-3’6183 (Rv) 5’-GGACGTAAACTCCTCTTCA-3’p53TOPO-cloning PCR product 6288/62896288 (FW) 5’-TAAGCAGCAGGAGAAAGCC-3’6289 (Rv) 5’-TTCTTTGCTGCCGTCTTCC-3’Sequencing with M13Fw
