# Supplementary material for: Spatial control of the APC/C ensures the rapid degradation of cyclin B1
Source: EMBO J. 2024 Aug 14;43(19):4324–55. doi: 10.1038/s44318-024-00194-2 (PMC11445581; doi:10.1038/s44318-024-00194-2)
Supplement: Supplementary file 13 — Expanded View Figures [file 44318_2024_194_MOESM13_ESM.pdf]

Expanded View Figures

Figure EV1. Characterisation of RPE-1 CyclinB1-mEmerald$^{+/+}$ and APC8-mScarlet$^{+/+}$ cells.

(**A**) Representative fluorescence confocal image of RPE-1 CyclinB1-mEmerald$^{+/+}$; APC8$^{+/+}$ cells in interphase. The scale bar corresponds to 20 µm. (**B**) Representative fluorescence confocal images over time of a CyclinB1-mEmerald$^{+/+}$; APC8$^{+/+}$ cell progressing through mitosis. Time is expressed as mm:ss. The scale bar corresponds to 10 µm. (**C**) Dot plots of the mitotic timing of parental RPE-1, RPE-1 APC8-mScarlet$^{+/+}$, RPE-1 CyclinB1-mEmerald$^{+/+}$ and APC8-mScarlet$^{+/+}$ cells, untreated (left) or treated with 100 nM paclitaxel (right). Each small dot represents one cell and large dots represent the median of independent experiments: $n \geq 83$ cells per condition, $N = 3$ independent experiments. Numbers on the graphs indicate the percentage of cells completing mitosis during the time of observation. Clones marked in red are the ones selected for all following experiments. (**D**) Top, growth curve of RPE-1 (black), RPE-1 APC8-mScarlet$^{+/+}$(orange), RPE-1 CyclinB1-mEmerald$^{+/+}$ and APC8-mScarlet$^{+/+}$(red), $N = 3$ experiment. Mean ± standard deviation are plotted. Bottom, dot plot of the chromosome number of parental RPE-1, RPE-1 APC8-mScarlet$^{+/+}$, RPE-1 CyclinB1-mEmerald$^{+/+}$ and APC8-mScarlet$^{+/+}$ cells. Each dot represents one chromosome spread: $n \geq 34$ spreads per condition, $N = 2$. (**E**) Representative anti-APC8, anti-APC4 and β-Tubulin immunoblot of cell lysates from parental RPE-1, RPE-1 APC8-mScarlet$^{+/+}$, RPE-1 CyclinB1-mEmerald$^{+/+}$ and APC8-mScarlet$^{+/+}$ cells before and after immunodepleting APC4, compared with control immunodepletion with IgG. (**F**) Bar graphs representing the quantification of the immunoblot in (panel **E**). $N = 2$ independent experiments. Mean ± standard deviation are plotted. (**G**) Graph representing the autocorrelation function of APC8-mScarlet over time in the nucleus. (**H**) Quantification of normalised Cyclin B1 fluorescence levels over time measured by spinning-disk fluorescence microscopy in RPE-1 CyclinB1-mEmerald$^{+/+}$ cells compared to RPE-1 CyclinB1-mEmerald$^{+/+}$; RPE-1 APC8-mScarlet$^{+/+}$, RPE-1 CyclinB1-mEmerald$^{+/+}$ cells. $n \geq 9$ cells per condition, $N = 3$ independent experiments. Mean ± standard deviation are plotted. Figure EV1 – Supplementary text to determine whether APC8-mScarlet is properly incorporated into the APC/C, we reasoned that immunoprecipitating an APC/C subunit would result in a depletion of APC8 levels in the lysate. Measuring the levels of APC8 immunodepletion following immunoprecipitation of APC4 revealed no significant cgq RPE-1, RPE APC8-mScarlet$^{+/+}$, and RPE APC8-mScarlet$^{+/+}$; Cyclin B1-mEmerald$^{+/+}$ cells, indicating that mScarlet does not interfere with APC8 incorporation into the APC/C.

© The Author(s)

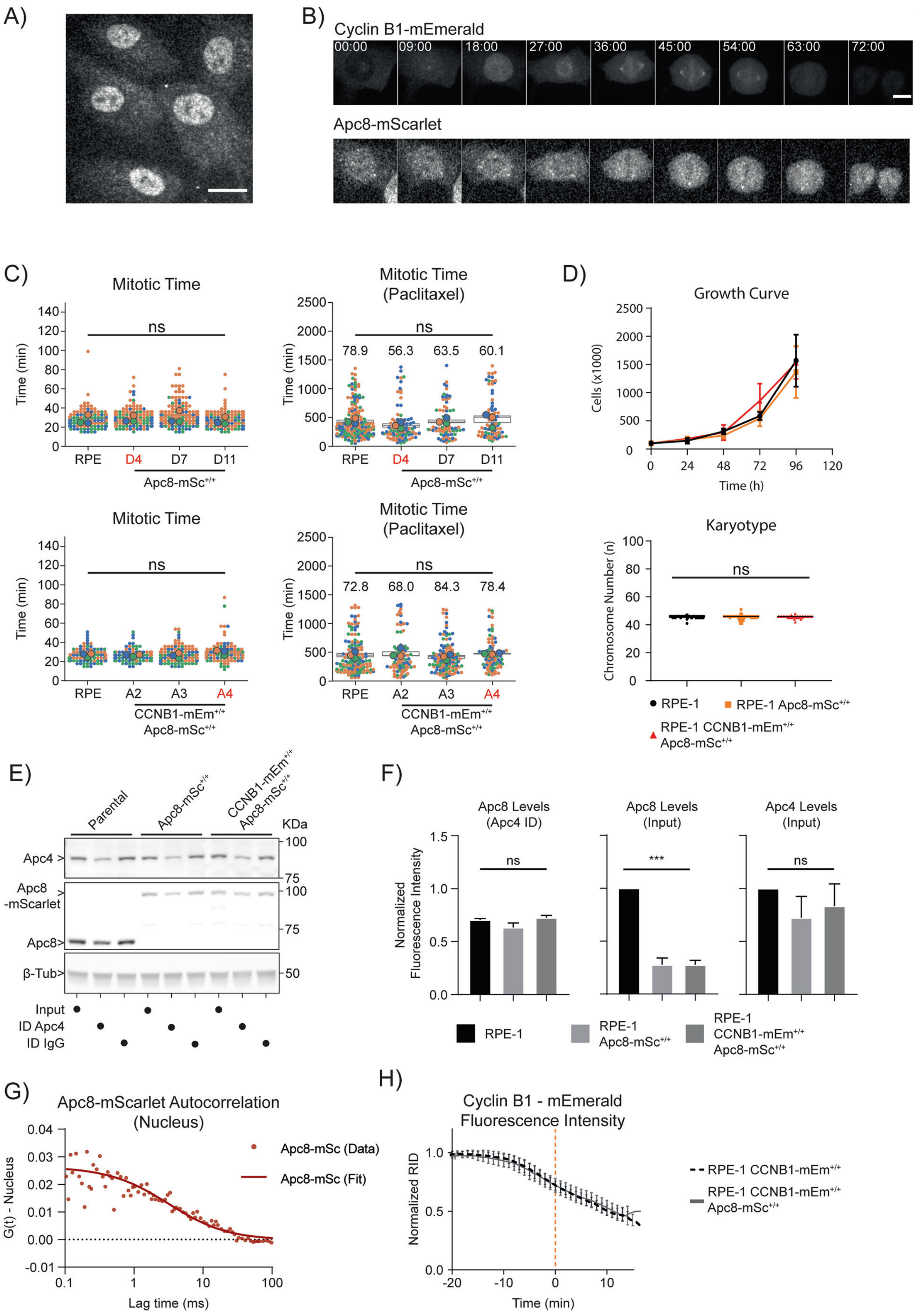
A)
B)
Cyclin B1-mEmerald
00:00 09:00 18:00 27:00 36:00 45:00 54:00 63:00 72:00
Apc8-mScarlet
C)
Mitotic Time
Time (min)
ns
RPE D4 D7 D11
Apc8-mSc+/+
Mitotic Time (Paclitaxel)
78.9 56.3 63.5 60.1
RPE A2 A3 A4
CCNB1-mEm+/+ Apc8-mSc+/+
72.8 68.0 84.3 78.4
D)
Growth Curve
Cells (x1000)
Time (h)
Karyotype
Chromosome Number (n)
RPE-1
RPE-1 Apc8-mSc+/+
RPE-1 CCNB1-mEm+/+ Apc8-mSc+/+
E)
Parental
Apc8-mSc+/+
CCNB1-mEm+/+ Apc8-mSc+/+
KDa
Apc4
Apc8 -mScarlet
Apc8
β-Tub
Input
ID Apc4
ID IgG
F)
Apc8 Levels (Apc4 ID)
Apc8 Levels (Input)
Apc4 Levels (Input)
Normalized Fluorescence Intensity
ns
***
G)
Apc8-mScarlet Autocorrelation (Nucleus)
G(t) - Nucleus
Apc8-mSc (Data)
Apc8-mSc (Fit)
Lag time (ms)
H)
Cyclin B1 - mEmerald Fluorescence Intensity
Normalized RID
Time (min)
RPE-1 CCNB1-mEm+/+
RPE-1 CCNB1-mEm+/+ Apc8-mSc+/+

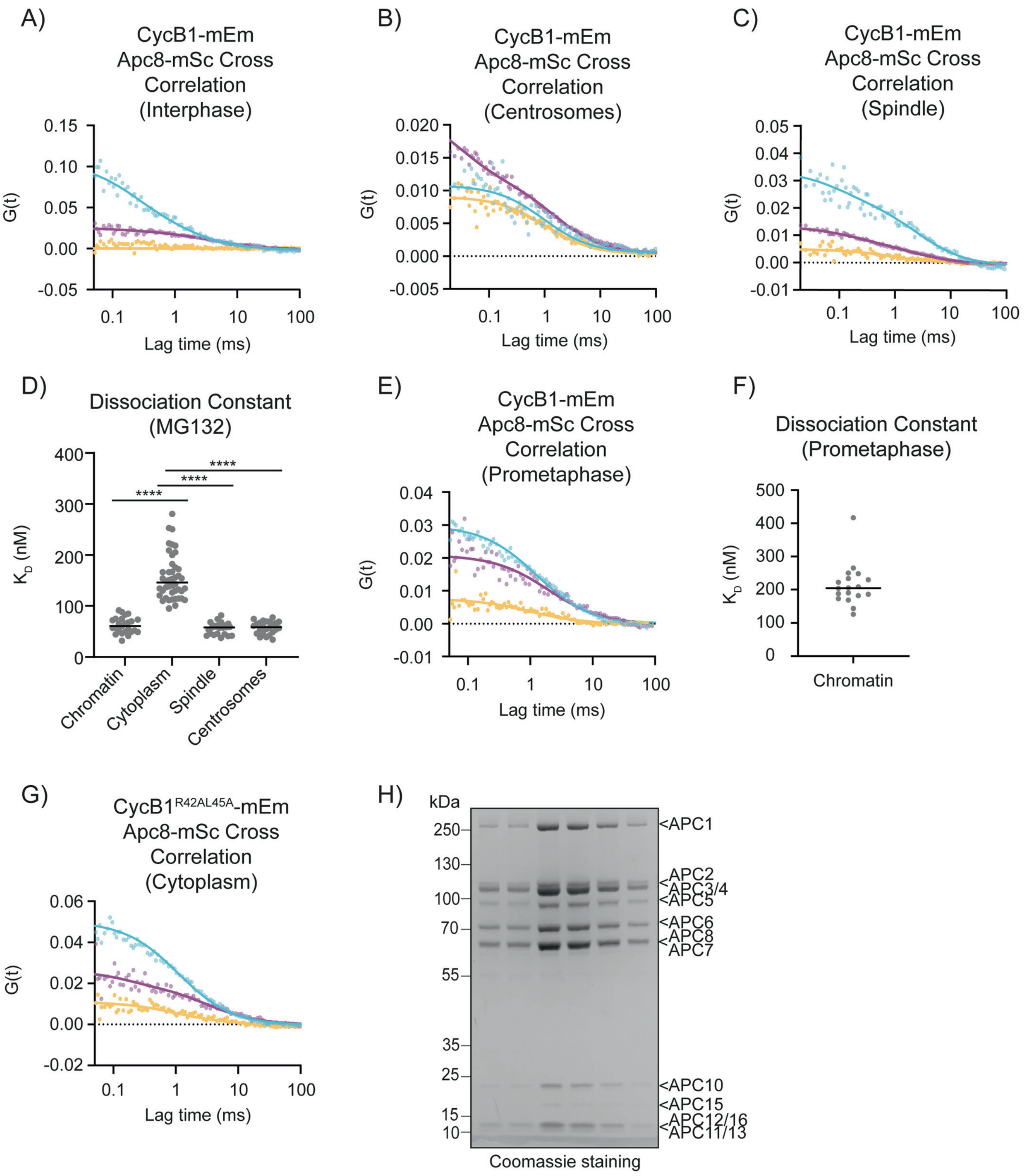

Figure EV2. FCCS of Cyclin B1-mEmerald and APC8-mScarlet.

(**A–C**, **E**, **G**) Representative graphs of the autocorrelation function of mEmerald and mScarlet and the cross-correlation function between the two in RPE-1 CyclinB1-mEmerald$^{+/+}$; APC8-mScarlet$^{+/+}$ (**A–C**, **E**), and in RPE-1 APC8-mScarlet$^{+/+}$ cells ectopically expressing Cyclin B1^{R45AL45A}-mEmerald (**G**). (**D**, **F**) Dot plots representing the K_D between endogenous Cyclin B1-mEmerald and APC8-mScarlet in metaphase cells following MG132 treatment (**D**) or in untreated prometaphase cells (**F**). $n \geq 18$ cells per condition, $N = 3$ independent experiments. (**H**) Fractions from a ResourceQ elution of the APC/C purification. Run on a 4–12% Bis-Tris SDS-PAGE gel.

 © The Author(s)

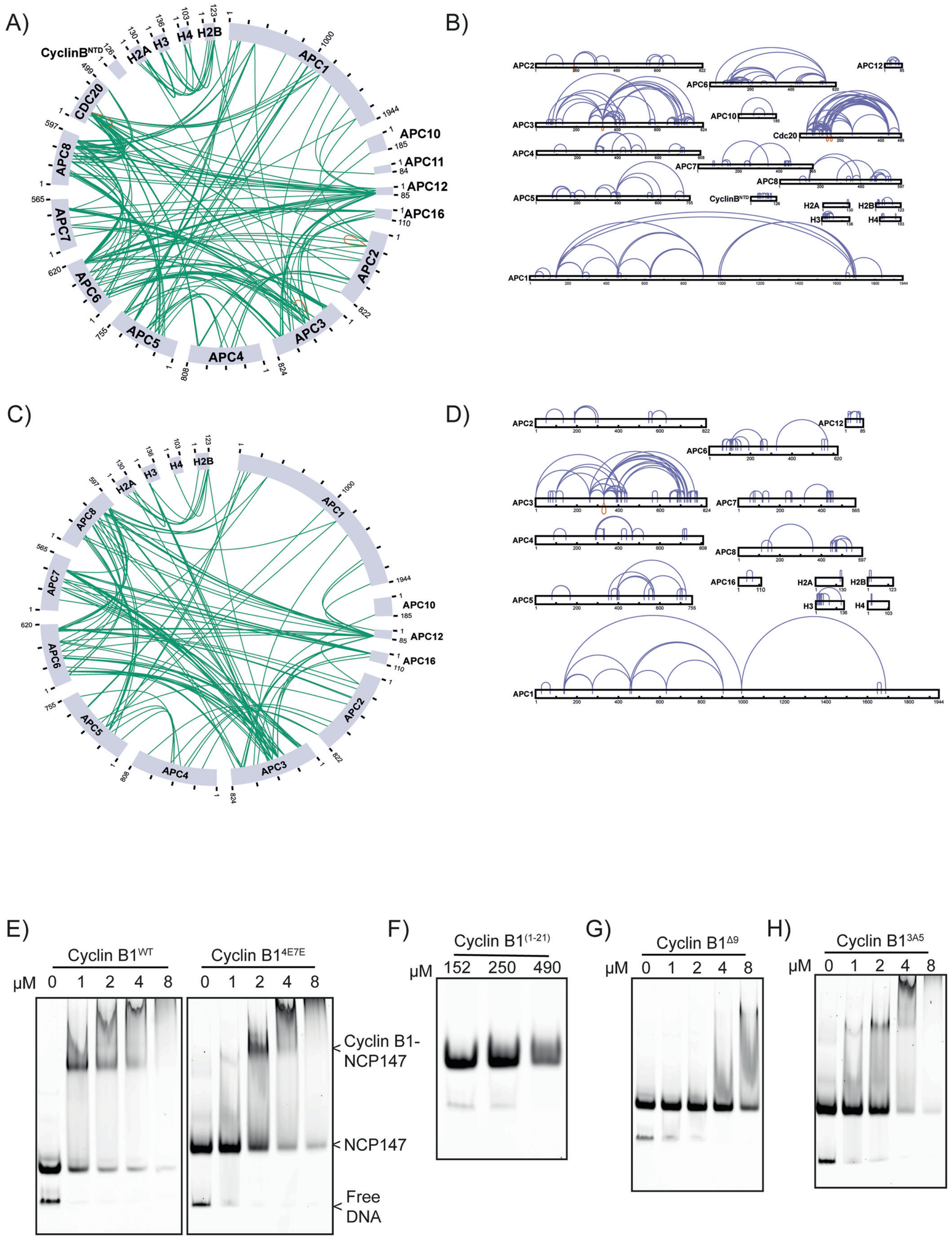
A)
B)
C)
D)
E)
Cyclin B1WT
Cyclin B14E7E
µM 0 1 2 4 8
Cyclin B1-NCP147
NCP147
Free DNA
F)
Cyclin B1(1-21)
µM 152 250 490
G)
Cyclin B1Δ9
µM 0 1 2 4 8
H)
Cyclin B13A5
µM 0 1 2 4 8

Figure EV3. Cyclin B1 binding to nucleosomes.

(**A**) Representation of heteromeric crosslinks of the APC/C-CDC20-Cyclin B1^{NTD} complex with the NCP. (**B**) Representation of the self-crosslinks of individual APC/C subunits, CDC20, Cyclin B1^{NTD} and the four histones. (**C**) Representation of heteromeric crosslinks of the APC/C with the NCP. (**D**) Representation of self-crosslinks of individual APC/C subunits and histones. (**E**–**H**). EMSA of NCP147 with the indicated variant of Cyclin B1. $N = 3$ independent experiments.

 © The Author(s)

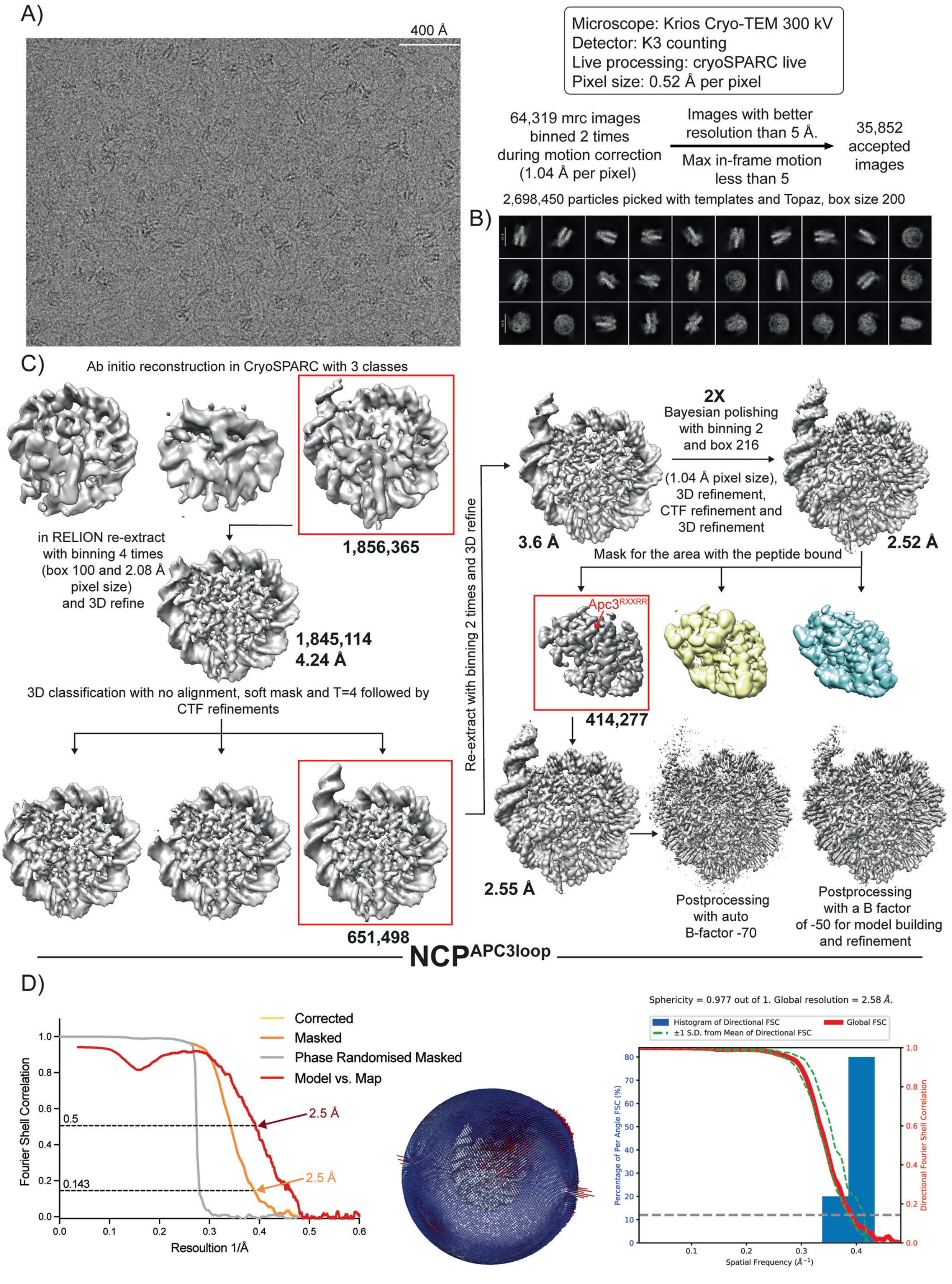

A)
400 Å
Microscope: Krios Cryo-TEM 300 kV
Detector: K3 counting
Live processing: cryoSPARC live
Pixel size: 0.52 Å per pixel
64,319 mrc images binned 2 times during motion correction (1.04 Å per pixel)
Images with better resolution than 5 Å.
Max in-frame motion less than 5
35,852 accepted images
2,698,450 particles picked with templates and Topaz, box size 200
B)
C)
Ab initio reconstruction in CryoSPARC with 3 classes
1,856,365
in RELION re-extract with binning 4 times (box 100 and 2.08 Å pixel size) and 3D refine
1,845,114
4.24 Å
3D classification with no alignment, soft mask and T=4 followed by CTF refinements
651,498
Re-extract with binning 2 times and 3D refine
3.6 Å
2X
Bayesian polishing with binning 2 and box 216
(1.04 Å pixel size), 3D refinement, CTF refinement and 3D refinement
2.52 Å
Mask for the area with the peptide bound
Apc3RXXRR
414,277
2.55 Å
Postprocessing with auto B-factor -70
Postprocessing with a B factor of -50 for model building and refinement
NCPAPC3loop
D)
Corrected
Masked
Phase Randomised Masked
Model vs. Map
Fourier Shell Correlation
Resoultion 1/Å
0.5
0.143
2.5 Å
2.5 Å
Sphericity = 0.977 out of 1. Global resolution = 2.58 Å.
Histogram of Directional FSC
±1 S.D. from Mean of Directional FSC
Global FSC
Percentage of Per Angle FSC (%)
Directional Fourier Shell Correlation
Spatial Frequency (Å⁻¹)

Figure EV4. Cryo-EM analysis of the nucleosome core particle in complex with APC3 loop$^{177\text{-}446}$.

(**A–C**) Workflow showing a representative micrograph, the cryo-EM data collection parameters (**A**) and the single-particle analysis pipeline for the nucleosome core particle with the APC3 loop$^{177\text{-}446}$ (**B**, **C**). N. of particles at each classification step is indicated. (**D**) Fourier shell correlation (FSC) curves, angular distribution plot and plot of the directional FSC that represent a measure of directional resolution anisotropy for all the reconstructions are shown. Directional FSC and sphericity determination was performed with the 3DFSC software (Tan et al, 2017).

 © The Author(s)

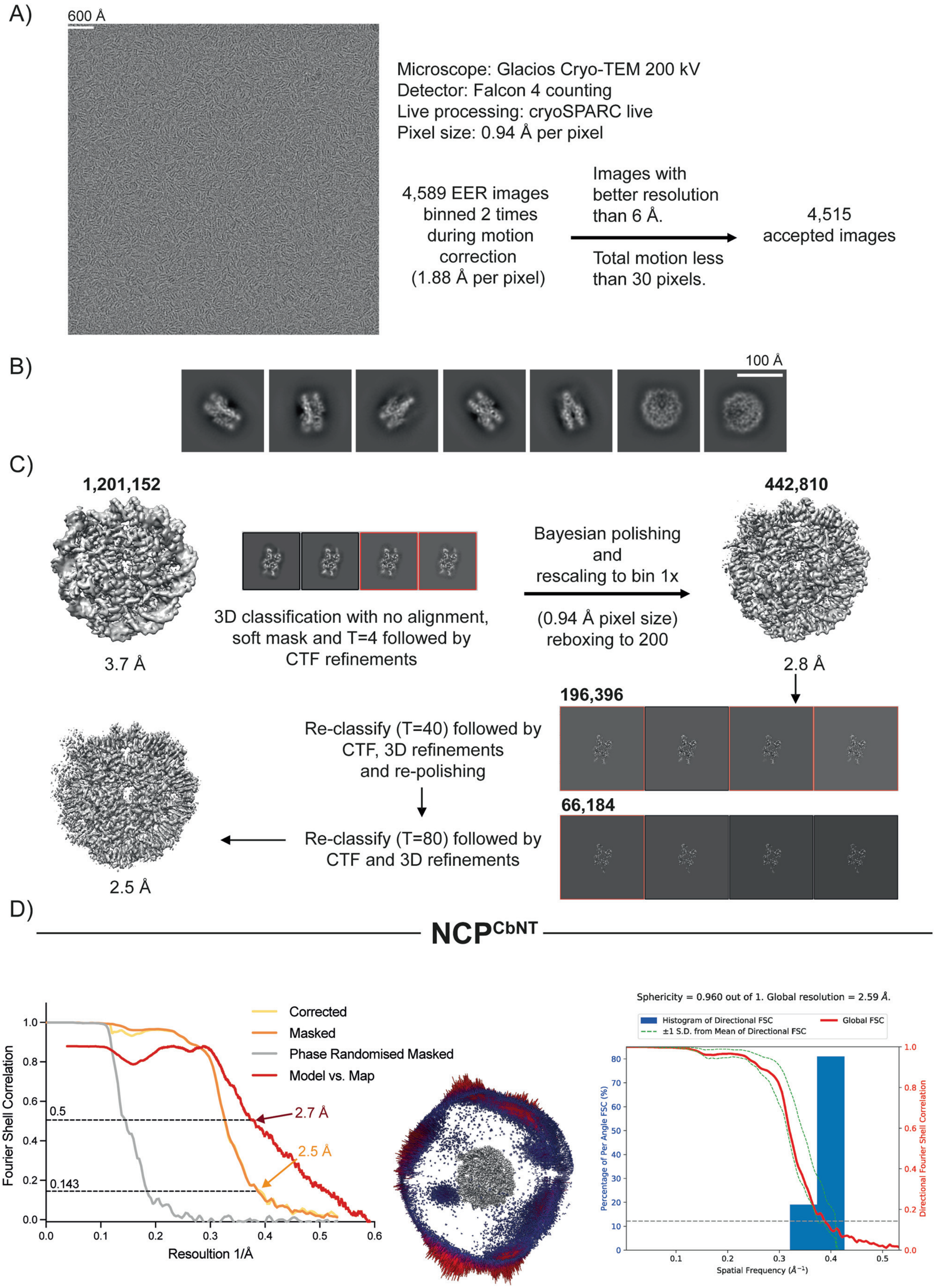
A)
600 Å
Microscope: Glacios Cryo-TEM 200 kV
Detector: Falcon 4 counting
Live processing: cryoSPARC live
Pixel size: 0.94 Å per pixel
4,589 EER images binned 2 times during motion correction (1.88 Å per pixel)
Images with better resolution than 6 Å.
Total motion less than 30 pixels.
4,515 accepted images
B)
100 Å
C)
1,201,152
3.7 Å
3D classification with no alignment, soft mask and T=4 followed by CTF refinements
Bayesian polishing and rescaling to bin 1x
(0.94 Å pixel size) reboxing to 200
442,810
2.8 Å
196,396
Re-classify (T=40) followed by CTF, 3D refinements and re-polishing
66,184
Re-classify (T=80) followed by CTF and 3D refinements
2.5 Å
D)
NCPCbNT
Corrected
Masked
Phase Randomised Masked
Model vs. Map
Fourier Shell Correlation
Resoultion 1/Å
0.5
0.143
2.7 Å
2.5 Å
Sphericity = 0.960 out of 1. Global resolution = 2.59 Å.
Histogram of Directional FSC
±1 S.D. from Mean of Directional FSC
Global FSC
Percentage of Per Angle FSC (%)
Directional Fourier Shell Correlation
Spatial Frequency (Å⁻¹)

Figure EV5. Cryo-EM analysis of the nucleosome core particle in complex with Cyclin B1 N-terminus.

(**A–C**) Workflow showing a representative micrograph, the cryo-EM data collection parameters (**A**) and the single-particle analysis pipeline for the nucleosome core particle in complex with Cyclin B N-terminus (NCP^{CbNT}) (**B**, **C**). N. of particles at each classification step is indicated. (**D**) Fourier shell correlation (FSC) curves, angular distribution plot and plot of the directional FSC that represent a measure of directional resolution anisotropy for all the reconstructions are shown. Directional FSC and sphericity determination was performed with the 3DFSC software.

 © The Author(s)
