# Supplementary material for: Spatial control of the APC/C ensures the rapid degradation of cyclin B1
Source: EMBO J. 2024 Aug 14;43(19):4324–55. doi: 10.1038/s44318-024-00194-2 (PMC11445581; doi:10.1038/s44318-024-00194-2)
Supplement: Supplementary file 12 — Appendix Figure Source Data [file 44318_2024_194_MOESM12_ESM.zip › SourceData/Appendix Figure 1.zip!/Appendix Figure 1/Fig S6A/README.rtf]

4643 = CycB1-mEm+/+ FRT CycB1WT-mSc
4645 = CycB1-mEm+/+ FRT CycB13A5-mSc
4646 = CycB1-mEm+/+ FRT CycB14E7E-mSc
4757 = CycB1-mEm+/+ FRT CycB1d(2-9)-mSc

C1 = mEmerald
C2 = mScarlet
C3 = siR-DNA
