# Supplementary material for: Spatial control of the APC/C ensures the rapid degradation of cyclin B1
Source: EMBO J. 2024 Aug 14;43(19):4324–55. doi: 10.1038/s44318-024-00194-2 (PMC11445581; doi:10.1038/s44318-024-00194-2)
Supplement: Supplementary file 12 — Appendix Figure Source Data [file 44318_2024_194_MOESM12_ESM.zip › SourceData/Appendix Figure 3.zip!/Appendix Figure 3/Fig S8A/README.rtf]

4869 = CycB1-mEm+/+ FRT CycB1d9—LANA-mSc


C1 = mEmerald
C2 = mScarlet
C3 = siR-DNA
